# The POM Monoclonals: A Comprehensive Set of Antibodies to Non-Overlapping Prion Protein Epitopes

**DOI:** 10.1371/journal.pone.0003872

**Published:** 2008-12-08

**Authors:** Magdalini Polymenidou, Rita Moos, Mike Scott, Christina Sigurdson, Yong-zhong Shi, Bill Yajima, Iva Hafner-Bratkovič, Roman Jerala, Simone Hornemann, Kurt Wuthrich, Anne Bellon, Martin Vey, Graciela Garen, Michael N. G. James, Nat Kav, Adriano Aguzzi

**Affiliations:** 1 Institute of Neuropathology, University Hospital Zurich, Zurich, Switzerland; 2 Functional Genomics Center Zurich, ETH/University of Zürich, Zürich, Switzerland; 3 Department of Agricultural, Food and Nutritional Science, University of Alberta, Edmonton, Alberta, Canada; 4 Department of Biochemistry, University of Alberta, Edmonton, Alberta, Canada; 5 Alberta Research Council, Edmonton, Alberta, Canada; 6 Department of Biotechnology, National Institute of Chemistry, University of Ljubljana, Ljubljana, Slovenia; 7 Faculty of Chemistry and Chemical Technology, University of Ljubljana, Ljubljana, Slovenia; 8 Institut für Molekularbiologie und Biophysik, ETH Zürich, Zürich, Switzerland; 9 Department of Molecular Biology and Skaggs Institute for Chemical Biology, The Scripps Research Institute, La Jolla, California, United States of America; 10 ZLB Behring, Marburg, Germany; Université de Toulouse, France

## Abstract

PrP^Sc^, a misfolded and aggregated form of the cellular prion protein PrP^C^, is the only defined constituent of the transmissible agent causing prion diseases. Expression of PrP^C^ in the host organism is necessary for prion replication and for prion neurotoxicity. Understanding prion diseases necessitates detailed structural insights into PrP^C^ and PrP^Sc^. Towards this goal, we have developed a comprehensive collection of monoclonal antibodies denoted POM1 to POM19 and directed against many different epitopes of mouse PrP^C^. Three epitopes are located within the N-terminal octarepeat region, one is situated within the central unstructured region, and four epitopes are discontinuous within the globular C-proximal domain of PrP^C^. Some of these antibodies recognize epitopes that are resilient to protease digestion in PrP^Sc^. Other antibodies immunoprecipitate PrP^C^, but not PrP^Sc^. A third group was found to immunoprecipitate both PrP isoforms. Some of the latter antibodies could be blocked with epitope-mimicking peptides, and incubation with an excess of these peptides allowed for immunochromatography of PrP^C^ and PrP^Sc^. Amino-proximal antibodies were found to react with repetitive PrP^C^ epitopes, thereby vastly increasing their avidity. We have also created functional single-chain miniantibodies from selected POMs, which retained the binding characteristics despite their low molecular mass. The POM collection, thus, represents a unique set of reagents allowing for studies with a variety of techniques, including western blotting, ELISA, immunoprecipitation, conformation-dependent immunoassays, and plasmon surface plasmon resonance-based assays.

## Introduction

Although the first anti-PrP antisera were first reported in 1984 [1], generation of high affinity monoclonal anti-PrP antibodies has been hindered for a long time by the fact that wild type mice are immunotolerant against PrP [2]–[4]. A widely used mouse monoclonal antibody, designated 3F4, was produced by immunizing mice with large amounts of purified SHaPrP^27-30^ [5]. 3F4 recognizes a hamster and human-specific PrP heptapeptide epitope, largely determined by methionine at positions 109 and 112 of hamster and human PrP, which is replaced with leucine and valine respectively in mouse PrP [6], [7]. However, a high level of PrP sequence conservation among species [8] limited the possibility of raising antibodies against species-specific epitopes and only a handful of high affinity antibodies were produced in wild type mice.

With the creation of *Prnp^o/o^* mice this obstacle was overcome [9], [10] and a number of very efficient monoclonal anti-PrP antibodies have been created by immunizing those mice with a variety of protocols from antibody-phage technology [11] to liposome preparations [12], DNA immunization [13], and recombinant protein [14], [15]. However, many of the currently available anti-PrP antibodies have specificities directed against the central and C-terminal part of the molecule. This results in part from the choice of antigen (purified PrP^27-30^) in most previous attempts to generate anti-PrP antibodies, from which the flexible unstructured N-terminal part of PrP is excluded.

We have developed and characterized a panel of novel anti-PrP monoclonal antibodies, with the goal of gaining new specificities towards PrP. These antibodies, designated POM1 through POM19, are directed against various epitopes spanning the entire sequence of the mature prion protein including the previously underrepresented N-terminal part. Some of those antibodies were previously shown to discriminate PK-digested PrP^Sc^ from different strains [16] thereby providing valuable tools for the biochemical characterization of prion strains [17] as well as for other aspects of prion research and diagnosis.

## Results

### Antibody production and selection

Using active immunization of *Prnp^o/o^* mice with bacterially produced recombinant mouse PrP (rmPrP_23-231_; numbering according to the human prion protein), we induced potent immune responses towards various protein-epitopes of PrP. Consequently, splenocytes of immunized mice were fused with the immortalized myeloma cell line Sp2/0-Ag14. Supernatants of growing clones were then screened for binding to rmPrP_23-231_ by Western Blot (WB) and ELISA. 55 positive clones were identified and were further analyzed by Western blot of brain homogenates from terminally scrapie sick mice (Supplementary Figure S1). This screen revealed several antibodies that performed well on western blotting, such as clones 75, 590, and 1193, henceforth named POM4, POM13, and POM1 respectively. Some other clones recognized mono and unglycosylated PrP, but not diglycosylated PrP^C^; these included clones POM4 (75), POM5 (141), and POM8 (248). Since the immunization was performed using bacterially produced rmPrP_23-231_ which is not glycosylated, the paratopes are unlikely to be directed against a glycan epitope. Instead it is likely that one of the two glycan chains sterically hinders the epitopes of those clones. Further experiments revealed that while these antibodies preferentially bind to mono and unglycosylated PrP, they also bind to diglycosylated molecules with reduced binding strength (e.g. in Supplementary Figure S4A).

Subsequently, all 55 clones were screened for binding to native PrP^C^ as it is displayed on the cell surface (Supplementary Figure S2). To this end, blood cells from transgenic mice overexpressing PrP specifically on T-cells [18] were used and all clones were compared directly to the previously described monoclonal antibody 6H4 [15] using flow cytometry. Many clones showed PrP-binding with fluorescent intensity of up to one log higher than that of 6H4. Based on this first screen of 55 positive clones, 19 most interesting clones were selected for further characterization, designated POM1–19 (Supplementary Figure S1 and S2, red frames).

### Isotyping and peptide mapping

Immunoglobulin (Ig)-isotype specific ELISA was performed using POM1–POM19. In a first screening round, plates were coated with N-terminally truncated recombinant mPrP comprising residues 121–231 (rmPrP_121-231_). After incubation with POM hybridoma supernatant, ELISA plates were incubated with HRP-labeled isotype-specific anti-mouse antibodies. Some POMs showed no binding on rmPrP_121-231_, indicating that their epitopes are located within the amino proximal part of PrP (data not shown). When full length protein was used for coating, those clones that were nonreactive with rmPrP_121-231_ yielded positive signals, and their Ig isotype was identified. Most of the POMs were found to be of the IgG_1_ isotype, with very few exceptions. This experiment allowed us to group the POMs into those reacting with epitopes either in the ‘unstructured’ N-terminal or in the structured PrP-domain 121–231 [16].

To further specify the epitopes of POM antibodies, a library of synthetic peptides spanning the whole PrP sequence was constructed. It consisted of 100 dodecameric peptides spanning PrP shifted by 2 amino acids. Using this library, competition-ELISA experiments were performed. To this end, plates were coated with rmPrP_23-231_ and incubated with POM-hybridoma cell supernatant in presence or absence of excess amounts of PrP peptides. Whenever the included peptide corresponded to the cognate epitope of the antibody, binding to rmPrP_23-231_ was reduced or abolished.

**Figure 1:**
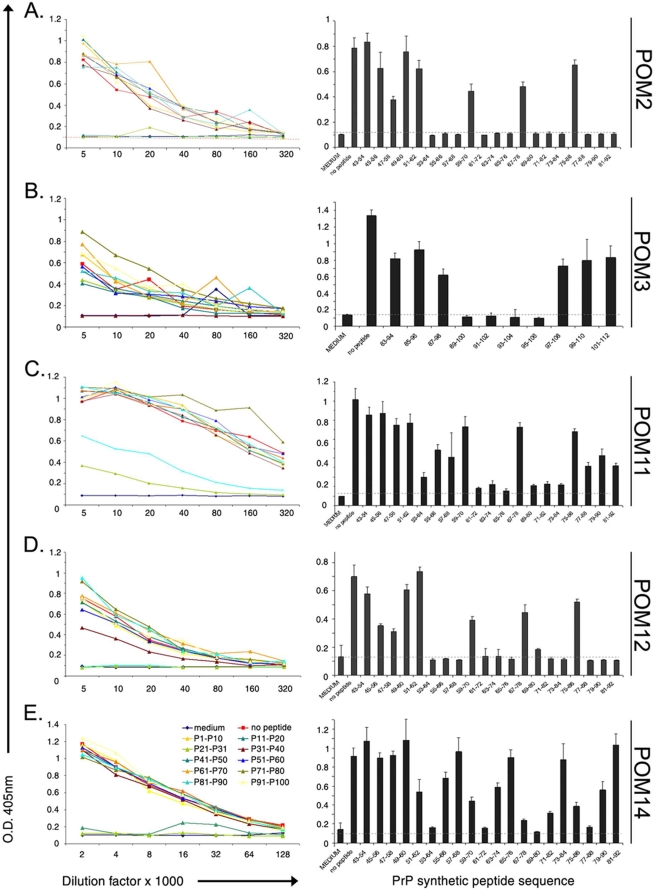
Competition ELISA and epitopes of N-terminal specific POMs. Competition ELISA with pools of 10 sequential peptides (all left panels) revealed a broad area of PrP including the epitope of each antibody; for example P11–P20 and P21–P30 for POM2 and P31–P40 for POM3. Subsequently, competition with single peptides (all right panels) representing the ones included in the competing pool from the left panel, identified the exact epitopes of POM2, 3, 11, 12 and 14. Note: although the right panels of A and D were previously published in the supplementary data of [16], we chose to include them here again for completeness.

Epitope mapping was performed in two steps. First, competition ELISA was performed using pools of 10 sequential peptides (Figure 1, all left panels). Inhibition of binding by one or two peptide pools exposed the PrP area that includes the epitope of each antibody. For example for POM2, peptides P11–P20 and P21–P30 competed meaning that the epitope is between amino acids 43–92 of mouse PrP. Subsequently, single peptides of the competing pool were used and the exact epitopes of POM2, 3, 11, 12, and 14 were identified (Figure 1, all right panels). Interestingly, of the five N-terminal antibodies mapped by this method, 4 have specificities on the octarepeat region of the PrP and only POM3 has an epitope downstream of the octarepeat region (HNQWNK, amino acids 95–100). This apparent increased immunogenicity of the octarepeat region is possibly due to its repetitive nature. POM2 and 12 seemed to recognize the same octapeptide, namely GQPHGGG/SW, which is located at 57–64, 64–72, 72–80 and 80–88 of mouse PrP and can carry either a glycine or a serine as the seventh residue. POM11 reacted mainly to octarepeats with serine on the seventh position, meaning amino acids 64–72 and 72–80 (GQPHGGSW); a similar sequence with glycine instead of serine showed lower competition efficiency (GQPHGGGW, residues 57–64 and 80–88). Finally, POM14 reacted with a 12-mer peptide resulting from two sequential octarepeats GGT/G/SWGQPHGGG/SW, scanning the overlapping PrP stretches 53–64, 61–72, 69–80 and 77–88. All octarepeat specific POMs have multiple epitopes on PrP. The POM2/12 epitope appears 4 sequential times on PrP, POM11 twice and POM14, 4 times that overlap twice, due to its length which is more than 8 amino acids.

**Figure 2:**
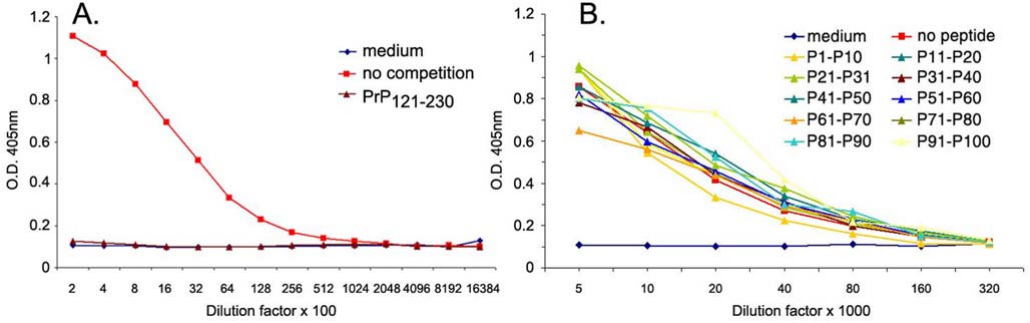
Linear 12-meric peptides do not compete with binding of C-terminal POMs to PrP. Binding of POM1 to immobilized rmPrP_23-231_ could be competed by excess amounts of N-terminally truncated rmPrP_121-231_ (A), but not with any of the PrP peptide pools (B) in contrast to all N-terminally specific POMs shown in Figure 1.

**Figure 3:**
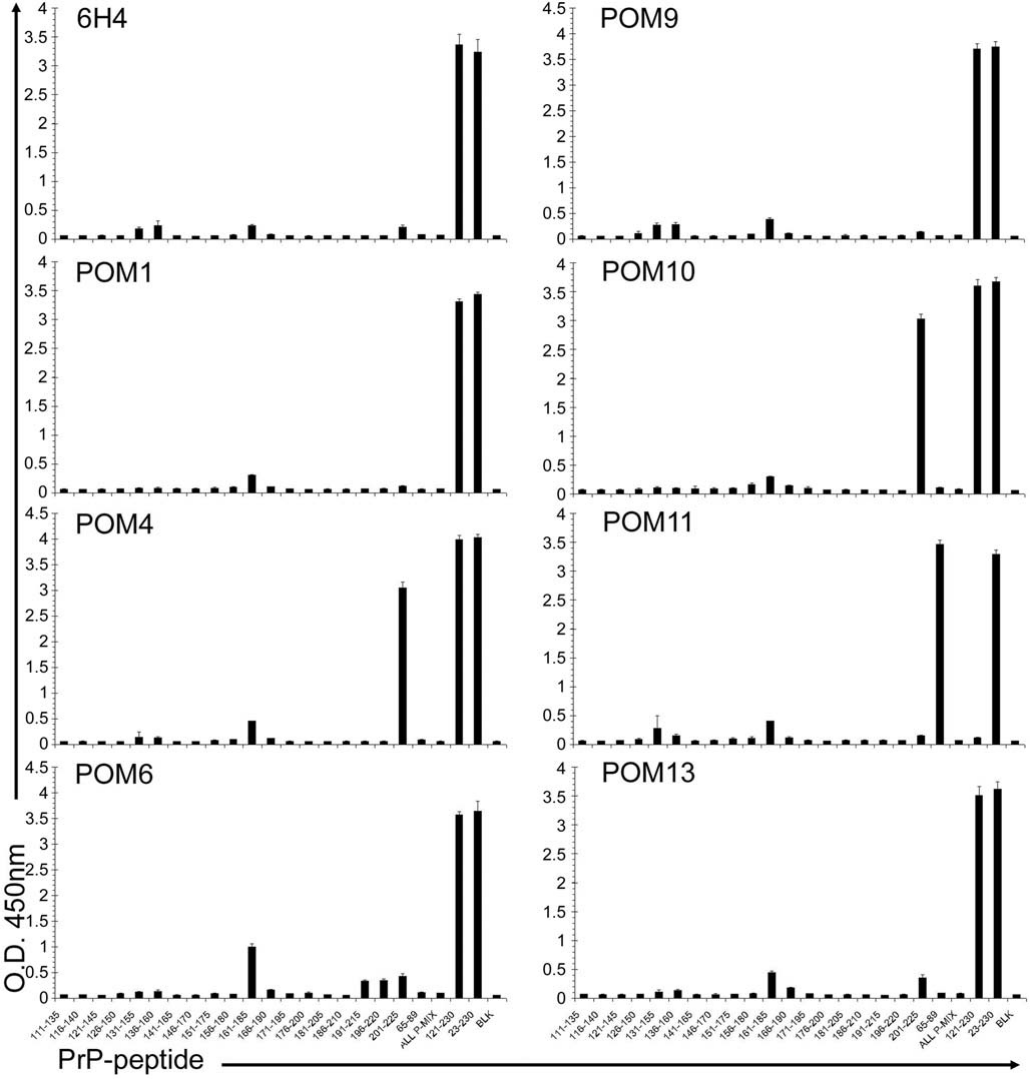
C-terminal POMs do not bind to linear 25-meric PrP-peptides. C-terminal-specific POMs were tested for binding on ELISA plates containing immobilized 25-meric PrP-peptides or rmPrP for control. While all antibodies bound strongly to rmPrP_23-231_, only POM4, 10 – and POM19, not shown – interacted with a 25-mer, spanning amino acids 200–225 of mouse PrP. As positive control, binding of POM11 on the octarepeat region – peptide 65–89 – was used. As expected POM11 did not interact with N-terminally truncated rmPrP_121-231_, while all other antibodies tested here did. When all peptides were mixed together and immobilized on the well (ALL P-MIX) no binding was observed, probably because of the inefficient amounts of the respective peptides. BLK: Blank.

Competition ELISA experiments were very informative for all N-terminal specific antibodies, but none of the C-terminal specific antibodies (binding on rmPrP_121-231_) could be mapped by this method (Figure 2). We then devised a panel of 19 longer synthetic peptides (25-mers) spanning the globular domain of the PrP sequence (amino acids 111–225) shifted by 5 amino acids each. The peptides were coupled on ELISA plates and binding of different POMs was assessed through a direct ELISA protocol. Full-length and N-terminally truncated rmPrP_121-231_ as well as a 25-mer spanning the octarepeat region were used as positive controls (Figure 3). While the octarepeat-specific POM11 clearly recognized both rmPrP_23-231_ and the octarepeat-mimicking peptides (amino acids 65–89), most C-terminal antibodies – with the exception of POM4 and POM10 – bound only rmPrP_23-231_ and rmPrP_121-231_ but none of the immobilized peptides. POM4, POM10 (Figure 3), and POM19 (not shown) bound a C-terminal peptide spanning amino acids 201–225, indicating that their epitopes are contained within that region. All other C-terminal antibodies tested however – including 6H4 – showed no or poor binding to the 25-mers and therefore gave limited information on their epitope specificity.

A very plausible explanation for this result is that POMs binding to the structured part of PrP identify either discontinuous or conformational epitopes that cannot be simulated by using short linear peptides. The complete segregation of short linear vs. long or discontinuous epitopes was entirely unexpected: not a single antibody to the structured region of PrP could be competed with linear dodecameric peptides, whereas all antibodies to the unstructured amino proximal region or to the very far carboxy terminal end of PrP, without any exception, were efficiently competed by cognate peptides.

### Pair-wise mapping by surface plasmon resonance

To overcome the epitope-mapping obstacles and to learn more about the binding specificities of the C-terminal POMs, surface plasmon resonance (SPR) technology was used in order to compile complementation groups of mAbs with non-competing epitopes. With this methodology it is possible to identify fully complementary mAb pairs that would not sterically hinder the binding of each other to the same protein, e.g. for establishing a highly sensitive sandwich ELISA for PrP^C^ and/or PrP^Sc^. Notably, this strategy was expected to function even if the exact epitopes of the antibodies are not known. In addition, competition experiments with 6H4, a previously mapped C-terminal anti-PrP antibody [15], could identify POM antibodies with epitopes in the vicinity of the 6H4 epitope (144–152 of mouse PrP).

**Figure 4:**
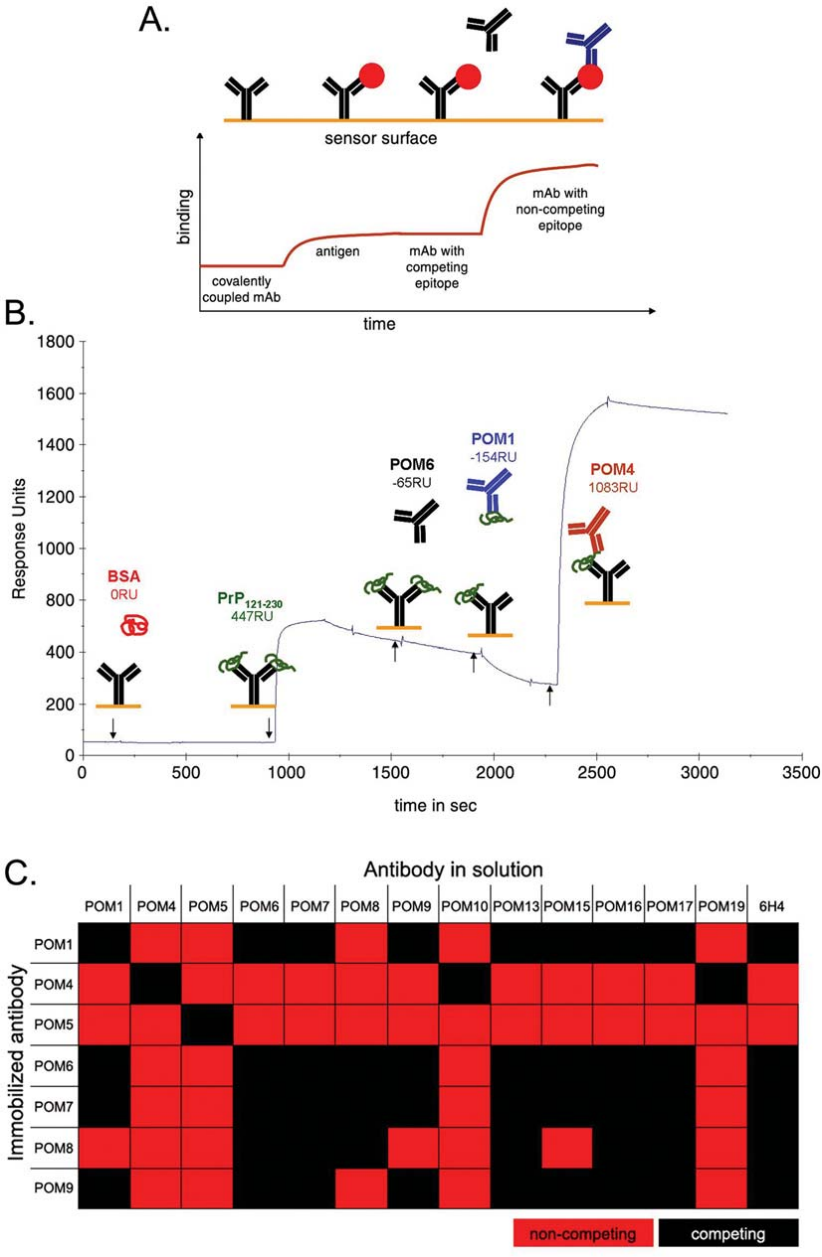
Pair-wise mapping of POM antibodies using surface plasmon resonance. (A) Schematic representation of experimental setup used pair-wise mapping. One antibody (shown in black) was immobilized on the chip surface and recombinant PrP (red circle) is injected into the system. Subsequently a second antibody (shown in blue) was injected and binding events were observed. (B) Representative pair wise mapping experiment with immobilized POM6. BSA is a negative control protein and POM6 re-injected into the system also serves as a negative control antibody (same antibody used for capturing PrP). RU: Response Units. (C) Summary of POM complementarity groups according to pair-wise mapping SPR-experiments.

In pair-wise mapping experiments, one antibody is immobilized on the chip surface by a covalent bond and captures recombinant mPrP that is consequently injected in solution. Next, a second antibody is injected and binding events are observed. If the second antibody has an epitope that competes with the capturing antibody it will not bind. On the contrary, if it has a non-competing epitope, it binds on PrP captured by the immobilized antibody (Figure 4A). A representative sensogram for immobilized POM6 is shown in Figure 4B. In this case, bovine serum albumin (BSA) was used as a negative control protein which did not bind to POM6. After capturing of rmPrP_121-231_, POM6 was injected again and, as expected, no binding was observed (same antibody = same epitope). Instead a gradual decrease in response units implies a slow dissociation of PrP. When POM1 was subsequently injected, no binding occurred on the chip surface. On the contrary, during POM1 injection the dissociation curve of rmPrP_121-231_ is augmented, indicating that POM1 is ‘stripping’ PrP from the POM6-coated surface. This suggests that POM1 binds an epitope that competes with POM6 for binding to PrP. POM4 is injected last and it shows a potent binding on the POM6-captured-PrP, demonstrating that it binds independently of POM6 epitope.

Similar sensograms were obtained for immobilized POM1, 4, 5, 6, 7, 8, and 9 with all other C-terminal POMs as well as 6H4 (data not shown). The results are summarized in Figure 4C. Pair-wise mapping of C-terminal POM antibodies performed by SPR demonstrated that all competing pairs of antibodies antagonized each other's binding in both orientations, independently of whether a mAb was immobilized or in solution, confirming the validity of these experiments. POM5 appears to have a unique epitope that can be combined with any other POM or with 6H4 without competition. POM4 competed only with POM10 and POM19, pointing out that these three antibodies cluster together, potentially sharing the same epitope. Antibodies POM1, 6, 7, 8 and 9 competed with 6H4 indicating that their epitopes lay in the vicinity of residues 144–152 of mouse PrP that correspond to 6H4 epitope. In addition, all C-terminal antibodies tested by pair-wise mapping on the SPR instrument, showed competition with themselves meaning that when PrP was captured by a particular antibody, the same antibody could not bind on PrP, when injected in solution. This was not surprising, since its epitope was blocked by the capturing moiety coupled to the chip surface. However, when the octarepeat specific antibodies POM2 and 12 were tested on the same setup, results looked different. As already mentioned, POM2 and 12 have epitopes that appear 4 sequential times on the N-terminus of PrP. This is probably why, when rmPrP_23-231_ is captured by POM12, some epitopes remain available for further binding when the same antibody is re-injected in solution [16].

POM1, 2, 3, 4, and 9 were biotinylated and used with their non-biotinylated equivalents for capturing and detecting recombinant mPrP in all possible combinations and orientations in a sandwich ELISA protocol (Supplementary Figure S3). As observed by SPR, POM2 was the only antibody that could be used simultaneously as capturing and detecting reagent (Supplementary Figure S3A–E). POM2 was also the most potent capturing antibody showing highest titers, independently of the detecting antibody (Supplementary Figure S3A–E). Furthermore, combination of POM1 and POM9 in either orientation gave only borderline signals (Supplementary Figure S3A and S3E), confirming competition of these two antibodies observed by pair-wise mapping in SPR (Figure 4C). POM4 does not compete with POM1, POM9 or the N-terminal POM2, as expected. Interestingly, POM3 seems to partially compete with POM4 only in one orientation, namely when POM4 is capturing PrP and POM3 is detecting it (Supplementary Figure S3C), but not in the reverse experiment (Supplementary Figure S3D). We think that this may be explained by the observation that a central part of PrP (residues 121–134) participates in the POM4 epitope (see below Figures 6 and S4). This domain is in the vicinity of the POM3 epitope and may therefore interfere with the subsequent POM3 binding.

### Epitope mapping by Western blotting and cross-species reactivity

**Figure 5:**
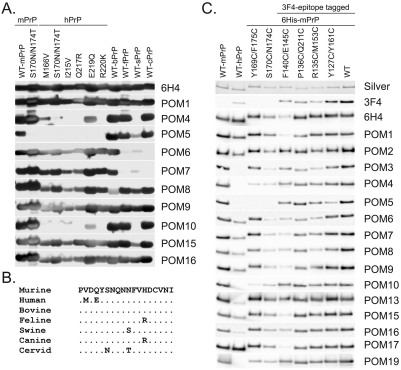
Cross-species specificities and binding idiosyncrasies of C-terminal specific POMs. (A) Western blot analysis on various wild type and mutated recombinant PrP proteins identified C-terminal POMs with particular binding patterns indicative for their epitopes (i.e POM4, 5, 6, 7 and 10). Blots were loaded with equal amounts and 6H4 serves as a loading control, since its epitope is conserved among the species tested and is not affected by the point mutations. (B) PrP sequence alignment of residues 165–182 demonstrating the position of POM5 epitope. (C) Western blot analysis on PrP variants, with various amino acids substituted by cysteine as indicated. Blots were loaded with equal protein amounts and a silver stained gel serves as a loading control (first panel). Only mild divergences in the binding patterns of the various POMs were found here. The most striking one is that 6H4, POM1 and POM17 are binding weaker to rmPrP with cysteine substitutions at positions 140 and 145.

To confirm and explore the grouping of C-terminal POMs that resulted from pair-wise mapping data and to gain further insight on their binding specificities and cross-species reactivity, a panel of wild type and point-mutated recombinant PrP proteins were tested by western blot. Considering the extremely high conservation of PrP across species it was fully anticipated that the POM collection includes mAbs reactive with PrP from other non-murine species. Indeed, most C-terminal antibodies i.e. POM1, 8, 9, 13, 15, 16 and 17 react with all wild type and mutant proteins, with intensities comparable to 6H4 (Figure 5A). Nonetheless, the remaining antibodies exhibited binding patterns reflecting their particular PrP-binding sites. For instance, POM4, 10 (Figure 5A) and 19 (not shown) present a common binding pattern, in line with data obtained by the SPR experiments (Figure 4C). These three antibodies do not bind to wild-type human and porcine PrP molecules, most likely due to sequence inconsistencies in residues necessary for forming the antigen-antibody bond. In addition, POM4, 10 and 19 do not bind to any of the point-mutants of human PrP molecules tested, except for the one carrying E219Q mutation (Figure 5A). Interestingly, this mutation represents a natural dissimilarity in the primary structure of human PrP versus most of the other mammals including mice; it is glutamic acid (E) in humans and glutamine (Q) in most other species. This indicates that glutamic acid at position 219 of human PrP is largely responsible for the fact that POM4, 10 and 19 do not bind it, since binding is regained when it is exchanged with glutamine, i.e. the mouse-equivalent residue. But porcine PrP also carries a glutamine at position 219, so why is it not recognized? Two residues away from the identified ‘hot-spot’ for POM4, 10 and 19 epitopes, at position 222, porcine PrP has a unique tyrosine (Y) instead of serine (S) in all other PrPs tested. This substitution is likely the reason for its incompatibility with the mouse PrP-developed antibodies. Taken together these results suggest that the epitopes of these three mAbs lay in the C-terminal part, approximately around amino acids 218 and 221 of the murine prion protein. Alternatively, substitution of these residues may result in structural changes that interfere with binding of POM4, 10 and 19 to PrP. POM6 and 7 faintly recognized porcine PrP but did not recognize feline and canine PrP. Sequence dissimilarities at positions 158 and 177 of feline and canine PrP in comparison to all other PrP molecules tested here may account for this. All antibodies except POM3 and POM5 were found to bind recombinant cervid PrP as well as cervid PrP^C^ from brain extracts (data not shown).

Another observation concerns POM5, which shows a unique binding pattern on several PrP molecules (Figure 5A), just like it showed a unique POM-competition pattern in the pair-wise mapping experiments (Figure 4C). POM5 does not bind to mouse PrP carrying two amino acid substitutions at positions 170 and 174 (S170N, N174T), suggesting that its epitope is located on this exact area of PrP. Failure of POM5 binding to the mutated mouse PrP was also confirmed by SPR (data not shown). POM5 also fails to detect human PrP, possibly due to sequence dissimilarity at position 168, which increases the negative charge of the protein at the binding site of POM5 possibly leading to destabilization of the antibody bond (Figure 5B). As observed at the first screen of the POM hybridoma supernatants, POM5 has an epitope that is sterically hindered by one glycan chain of PrP (Supplementary Figure S1). Based on the predicted localization of POM5 epitope in the area of 168–174 of mouse PrP, this is most likely the glycosylation linked to asparagine 181. Figure 5B shows a sequence alignment of PrP^C^ molecules from different species on the position of the estimated POM5 epitope.

We went on to test a second panel of mutant PrP proteins, in which various amino acids were substituted by cysteines. We have used a silver stain to ensure that the proteins were loaded at similar levels and we then compared replica blots looking for binding patterns that reveal information on the specificity of each antibody (Figure 5C). Surprisingly the differences in the binding patterns were much less prominent when compared to panel 8A. Nonetheless, we were able to determine that mutations at positions 140 and 145 of mouse PrP strongly reduced the binding of 6H4, POM1 and POM17, indicating that these positions are significant for the epitopes of these antibodies. In the case of 6H4, this is in accordance with previously published data, since the glutamic acid at position 145 lies within the published epitope [15]. In addition, antibodies POM6–POM9 showed reduced binding to both 170/174 and 140/145 cysteine mutants. Since these two mutants have cysteine substitutions at distant positions of the protein, the apparent reduction in the binding suggests either that the epitopes of POM6–POM9 are discontinued or conformational. Confirming our previous results, antibodies POM3, POM4, POM5, POM10 and POM19 did not recognize human PrP (Figure 5C).

**Figure 6:**
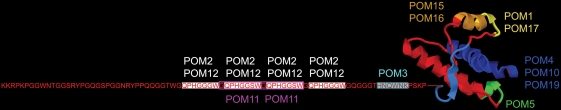
Summary of epitope specificities of POM antibodies. POM2 and 12 bind a degenerative epitope within the octarepeat sequence, whereas POM11 shows stronger binding to a mouse-specific variation of the sequence – QPHGGSW instead of QPHGGG/SW for the other two. POM3 binds a unique sequence near the centre of the PrP molecule (aa 95–100: HNQWNK). The rest of the POMs bind epitopes within the globular domain of PrP^C^ and were only approximately defined.

Further investigation of the C-terminal-specific POMs was achieved using brain homogenates of transgenic mice expressing N-terminally truncated PrP variants, designated ΔE with deletion of 33–121 amino acids and ΔF with a longer deletion (33–134) which includes the hydrophobic central domain of PrP [19]. Results confirmed the specificities of N-terminal POMs, which did not bind to ΔE PrP (Figure S4A). In addition, POM5 and POM7 only recognized un- and monoglycosylated PrP bands, due to their ‘glycosylation-sensitive’ epitopes. In contrast to all other POMs tested, POM2 and 12 identified a protein in *Prnp^o/o^* brain homogenates. This unknown protein of about 37kDa cross reacts with POM2/12 presumably because of a high degree of identity to the epitope contained in PrP (Supplementary Figure S4A). All C-terminal specific antibodies recognized ΔE-PrP, confirming results obtained from direct ELISA experiments. Unexpectedly, POM4 and 10 antibodies (and POM19, not shown), did not bind to ΔF PrP, in contrast to the rest of the C-terminal-specific POMs (Supplementary Figure S4B). This indicated that PrP residues 121–134 represent an indispensable component for binding of POM4, 10 and 19. Conversely, western blots of mutated PrP proteins indicated that amino acids 219 and 222 are essential for POM4, 10 and 19 interaction with PrP (Figure 5A). Taken together, these results suggest that these antibodies recognize a conformational epitope, consisting of the hydrophobic domain 121–134 and 201–225 of mouse PrP. Alternatively, deletion of this N-terminal part may result in a conformational change in PrP leading to the masking of the C-terminal bona fide epitope of POM4, 10 and 19. PrP residues 121–134 are perfectly conserved among species. Since POM4, 10 and 19 do not bind human but only mouse PrP, their epitope must be somehow disrupted in human PrP-structure, possibly through 201–225, its C-terminal counterpart. The epitopes of the POM antibodies are summarized in Figure 6.

### Flow cytometry, immunohistochemistry and immunoprecipitation

**Figure 7:**
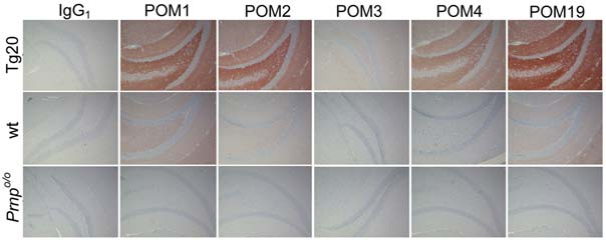
Immunohistochemistry on mouse brain for staining of PrP^C^. Consecutive brain sections of Tg20, WT, or *Prnp^o/o^* mice focusing near the hippocampus area were incubated with the same concentration of each POM or IgG_1_ control antibody. The same HRP-conjugated anti-mouse antibody was used for development, allowing direct comparison of the performance of each antibody. POM1 and POM19 seemed to be the most sensitive antibodies in this assay, since they could readily detect PrP^C^ in the wild-type brains.

Purified POM antibodies were systematically tested for binding on PrP^C^ by flow cytometry, immunohistochemistry, and for their capacity to immunoprecipitate PrP^C^ and PrP^Sc^. Performance of each POM on flow cytometry was compared to that of 6H4 [15], which in the past had been often used for visualizing PrP^C^ by flow cytometry. PrP-overexpressing T-cells [18] from blood were stained in two steps using purified POMs or 6H4 in equal concentrations and fluorescently labeled anti-mouse IgG, in order to compare binding intensities of all mAb as previously described [4]. Direct comparison to 6H4 with normalized concentrations of all mAbs revealed that all N-terminal specific POMs bind very strongly to native PrP as displayed on the surface of live cells (Supplementary Figure S5). Immunohistochemistry (IHC) on paraffin embedded brain sections of wild type, *Prnp^o/o^* and *Tg20* (PrP-overexpressing) mice identified POMs performing extremely well when a standard PrP^C^-staining protocol was used (Figure 7). However, none of the POMs were reactive with PrP^Sc^ from prion infected mouse brain (data not shown).

**Figure 8:**
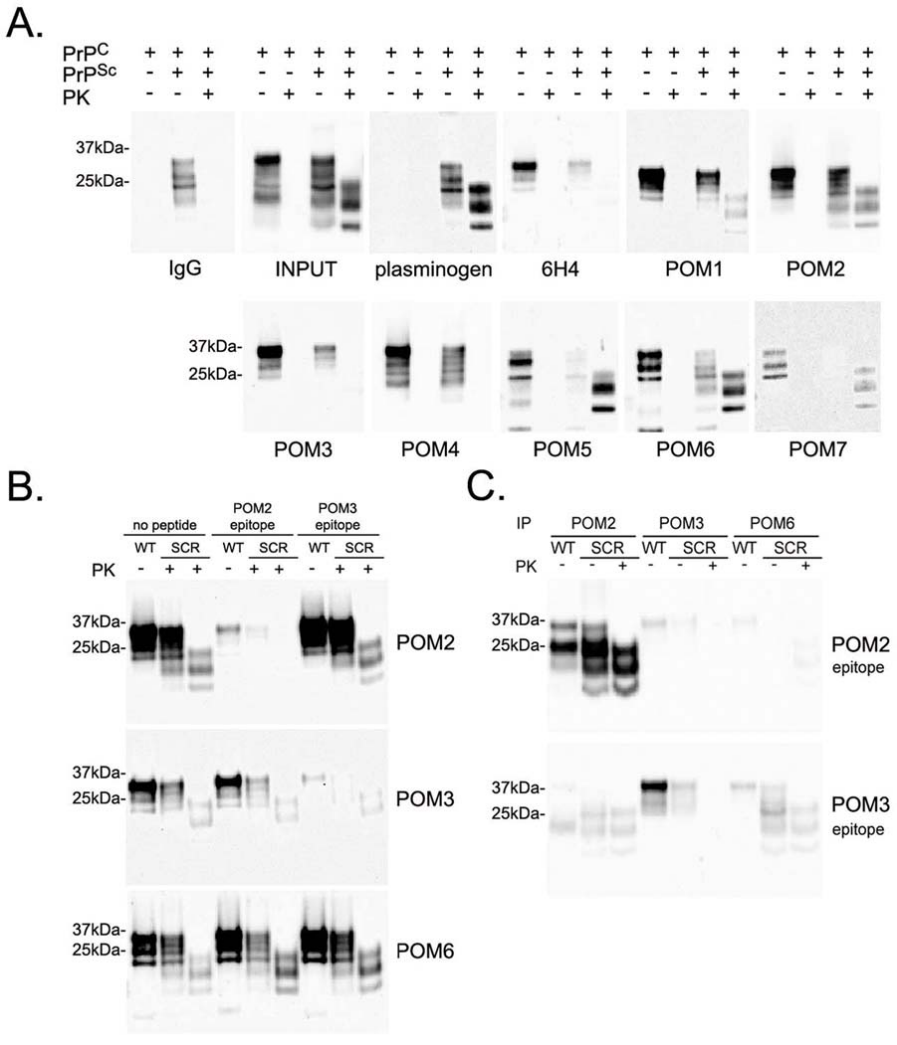
Immunoprecipitation, peptide competition and specific elution of PrP^C^ and PrP^Sc^ with POMs. (A) Brain homogenates from healthy or terminally scrapie sick mice were incubated with POM1–7, covalently coupled to paramagnetic beads. All antibodies tested immunoprecipitated PrP^C^ very efficiently and POM2, 5 and 6 showed competent immunoprecipitation of PK-digested PrP^Sc^. Plasminogen was used as a positive and non-specific IgG as a negative control. (B) Brain homogenates from healthy or terminally scrapie sick mice were mixed with POM2-, POM3- or POM6-coupled beads, which were pre-incubated with a peptide corresponding to the epitopes of POM2 (QPHGGGW) or POM3 (HNQWNK) or without any peptide for comparison. Pre-incubation of POM2-coupled beads with its epitope blocked immunoprecipitation of both PrP^C^ and PrP^Sc^, whereas same amounts of an unrelated peptide (POM3-epitope) had no effect on the binding. Similarly, immunoprecipitation of PrP^C^ by POM3 was only blocked by its corresponding peptide-epitope and not by the epitope of POM2. As expected none of the peptides interfered with binding of POM6 which interacts with the C-terminal part of PrP. SCR: scrapie. (C) PrP^C^ and PrP^Sc^ can be eluted from POM2-coupled beads after incubation with excess amounts of POM2 – but not POM2 – peptide epitope. Conversely PrP^C^ is found in the eluates of POM3-coupled beads when they are incubated with POM3 – but not POM2 – peptide epitope. Eluates from POM6-coupled beads show only trace amounts of both PrP isoforms. All membranes were immunoblotted with biotinylated POM1 and HRP-labeled avidin.

Binding of POM1–7 to PrP^C^ and PrP^Sc^ was also tested using a standard immunoprecipitation protocol (Figure 8A). Brains from healthy or terminally scrapie sick mice were homogenized and then incubated with POM1–7 covalently coupled to paramagnetic beads as previously described [20]. Precipitated PrP^C^ and PrP^Sc^ were visualized by western blot using biotinylated POM1 and HRP-labeled avidin. As expected, POM1–7 immunoprecipitated PrP^C^ from uninfected brain homogenate. However precipitation of PrP^Sc^ was more difficult to evaluate. Plasminogen has been previously shown to quantitatively immunoprecipitate PrP^Sc^ [20] and was therefore used under identical conditions as positive control and purified mouse IgG (unspecific) served as negative control. As recently described, PrP^Sc^ interacts with unspecific immunoglobulins possibly with their Fc moiety [21]. This was observed here in scrapie infected non-PK-digested brain homogenate. However, when the infected homogenate was digested with PK, no interaction was observed, allowing evaluation of antibodies binding to PK-digested PrP^Sc^. POM2, 5 and 6 displayed efficient immunoprecipitation of PK-digested PrP^Sc^ (Figure 8A). Whereas the POM2 epitope lies within the region of PrP that is digested by PK, at least one POM2 epitope remains in the PK-digested PrP^Sc^ from RML as evidenced by the fact that PrP^Sc^ retains POM2 immunoreactivity after incubation with PK (Supplementary Figure S1). In CJD type 2 and in further prion strains, however, the PK cleavage site lies downstream the complete POM2 epitope, thereby rendering PK-digested PrP^Sc^ ‘invisible’ to POM2 [16].

Pre-incubation of POM2-coupled beads with a peptide corresponding to its epitope blocked immunoprecipitation of both PrP^C^ and PrP^Sc^, providing evidence that POM2 binding occurs through the PrP-specific pocket of the antibody and not by any unspecific interactions (Figure 8B).

We then tested whether the POM2 epitope-mimicking peptide could be used to compete for the binding of PrP^C^ and PrP^Sc^ after it is bound on the POM2-loaded beads. This could allow for specific elution of the PrP isoforms and potentially other proteins specifically binding to them. We repeated the immunoprecipitation of PrP^C^ and PrP^Sc^ with POM2, POM3 and POM6 and then incubated the beads with excess amounts of POM2 or POM3 epitope-mimicking peptide. We found that the POM2 epitope could efficiently elute PrP^C^ and PrP^Sc^ from the POM2-coated beads, but not from beads coated with POM3 or POM6 (Figure 8C, upper panel). Conversely, the POM3 epitope eluted PrP^C^ from POM3-coated beads, while only traces of PrP^C^ and PrP^Sc^ were detected in the eluates from POM2- or POM6- coated beads, confirming the specificity of the elution protocol (Figure 8C, lower panel).

### Isolation of POM scFv

We went on to construct single chain antibodies (scFv) from POM2, 11, 18 and 19 hybridoma cells with the goal to isolate and sequence their heavy and light antibody chains. Moreover, the scFv POMs enabled experiments to be performed for which IgGs may not be optimal, such as immunohistochemistry, affinity determinations and *in vivo* passive immunization experiments.

In order to isolate antigen-binding scFv of POM2, 11, 18 and 19, at least 3 rounds of panning were required and the sequences of several good binders for each POM antibody were determined. Based on phage ELISA results, the strongest binders were isolated and used for subsequent expression and purification of POM scFv. The absorbance values (above background) for the strongest binders were: POM2 = 1.416, POM11 = 0.654, POM18 = 0.958 and POM19 = 0.306. Although there were other clones that were identified as strong antigen binders for each of the POMs, the sequences for many of them were found to be similar (data not shown), which was expected since the scFv genes were derived from hybridoma (monoclonal) cells. An amino acid sequence alignment comparison of the POM scFvs (the best binders) is shown in Supplementary Figure S6. While the sequences are similar, particularly for the N-terminal specific scFvs (POM2, 11 and 18), there are a number of amino acid differences in the complementarity determining regions (CDR) as well as in the flanking regions which may account for the differences in binding properties of their original monoclonal antibodies.

### Expression and purification of POM2 scFv

In order to characterize POM2 scFv in detail and compare the properties of this recombinant antibody with its parent monoclonal antibody, we overexpressed its cDNA in *E. coli* and purified the recombinant scFv. The expression of POM2 scFv was found to be almost exclusively within the insoluble inclusion bodies, which necessitated the use of denaturing conditions during purification followed by a refolding dialysis step. Supplementary Figure S6B shows the results of the SDS-PAGE analysis of the purified POM2 scFv. There appears to be no contaminant protein bands visible in the elution fraction lanes in the Coomassie Brilliant Blue R250-stained 13% SDS-PAGE gel indicating a relatively high degree of purity. During the refolding of the POM2 scFv, a large amount of precipitation was observed and the degree of precipitation did not appear to be affected by the concentration of POM2 scFv being dialyzed. Despite the precipitation of scFv, the yield of refolded, fully functional POM2 scFv was determined to be approximately 10–15mg/L of bacterial culture. The purified, refolded POM2 scFv was used in an ELISA with the N-terminal PrP peptide and it was observed to be functional (data not shown). The functional POM2 scFv was used in further studies aimed at characterizing this scFv.

### Affinity determination of selected POMs

In the last part of our study, we have adapted a previously described methodology [22] that utilized SPR technology to determine dissociation constants (*K_D_*) of selected antibodies. We immobilized recombinant rmPrP_121-231_ (or BSA as negative control) on the chip surface through a covalent bond, making sure that a low density PrP-covered plane is created (less than 1000 Resonance Units). We then injected POM19 at various increasing concentrations, ranging from 420 pM to 110 nM, regenerating the chip after each injection. We monitored the association and dissociation behavior of each solution (Supplementary Figure S7A) and we fitted the curves using algorithms available in the BIAevaluation 4.1 software. Based on these measurements, we have calculated that the binding constant of POM19 is 870 pM. Following a similar procedure we found the binding constant of POM1 to be 580 pM (data not shown).

When we followed the same protocol to calculate the affinities of the octarepeat-specific antibodies, however, we did not obtain curves that could fit in any of the available models of the BIAevaluation software. This was probably due to the fact that the reaction stoichiometry became increasingly complex, since we tested the binding of the bivalent IgG (POM2 or POM12) antibody to the tetravalent rmPrP_23-231_ – since the POM2 and POM12 epitope occurs four times in the PrP sequence. In order to overcome this obstacle, we attempted to calculate the equilibrium dissociation constants with competition BIAcore [23]. In this method, the BIAcore is used merely as a sensitive instrument to measure free antibody concentrations. Using a panel of solutions of defined antibody concentrations – ranging from 12nM-125pM – we first created a standard curve of POM2 IgG (data not shown) or scFv (Supplementary Figure S7B). We then prepared a 6nM POM2 scFv solution and mixed it with a series of defined concentrations of the epitope-mimicking peptide (13 different concentrations, ranging from 100-0.01nM). The solutions were allowed to reach equilibrium and then the concentration of free scFv POM2 in each tube was measured by BIAcore. We plotted the values of free antibody against those of the peptide competitor using the BIAevaluation software (Supplementary Figure S7C). This allowed for an estimation of the single binding event of the two monovalent reagents, scFv POM2 to the singular peptide epitope, is 20nM.

The actual binding of the bivalent IgG POM2, however, is a result of the cooperative interaction of each paratope of the IgG molecule with two – either consecutive or intermittent – epitope stretches within the octarepeat region of PrP. This translates in an apparent binding strength that is much higher and is the most probable reason why POM2 and POM12 are among the most sensitive antibodies of our series in most assays tested. In an attempt to determine the avidity of the reaction: POM2+2PrP → POM2PrP_2_ through theoretical calculations, we assumed that the two sub-reactions POM2+PrP → POM2PrP and POM2PrP+PrP → POM2PrP_2_ are defined by equal K_D_. This assumption allows for a simple calculation of the avidity of the total one-to-two reaction as follows: if K_D1_ = K_D2_ = K_D_, then K_Dtotal_ = K_D_^2^ = (2*10^−8^)^2^ = 4*10^−16^M^2^.

## Discussion

Here, we report the production and detailed characterization of a panel of monoclonal antibodies against PrP, designated POM1–POM19. These antibodies were raised against recombinant mouse rmPrP_23-231_ in PrP-deficient (*Prnp^o/o^*) mice and selected for binding to both denatured PrP in Western Blot (Supplementary Figure S1) and native, cell-bound PrP^C^ by flow cytometry and ELISA (Supplementary Figure S2).

We had little success in mapping the epitopes of the POM antibodies using linear short peptide arrays. We could, however, identify the epitopes of all antibodies that bind within the first 100 amino acids of PrP by peptide competition ELISA. We found these data to be more informative, since they could automatically be translated into a tool for testing the binding specificity of immunoprecipitation results and other assays (Figure 8B).

Additionally, the competing peptides could be used as specific elution reagents for PrP^C^ and PrP^Sc^ (Figure 8C). This is extremely useful since it has recently been found that, due to the hydrophobic nature of exposed regions of PrP^Sc^, the specificity of immunoprecipitation reactions should be treated with caution [21]. Our results provide a method that allows for a direct test for specificity of PrP^C^ and PrP^Sc^ immunoprecipitation, as well as for specific elution of both isoforms and their interacting partners. While the physiological role of PrP^C^ and the toxic mechanism of PrP^Sc^ remain mysterious, future proteomic analysis of such specific, PrP-containing eluates may shed some light on these important issues.

Why were no peptide competitors found for any of the antibodies interacting with the globular domain of the PrP molecule? This unexpected result was most likely due to these antibodies recognizing discontinuous or conformational epitopes. The latter hypothesis is not formally excluded, but seems highly unlikely since all POM antibodies recognized PrP on Western blots under denaturing conditions. Although under western blotting conditions proteins are denatured prior to transferring to a membrane, refolding of the protein and of ‘conformational’ antibody epitopes on the membrane can occur. The reactivity of a monoclonal antibody in a western blot format does therefore not definitively exclude its reactivity with a structured epitope.

Only a small fraction of monoclonal antibodies can be mapped with linear peptides, whereas most antibodies are best mapped using mutant or truncated versions of the antigen. In our analysis, indirect information about the binding sites of some of our antibodies was gained through this approach (Figure 5 and S4). For example POM4, 10, and 19 were found to bind a discontinuous epitope consisting of a very C-terminal part of the protein (around amino acids 220) and a middle part of the protein at amino acids 121–134. The latter was indentified, because these three antibodies did not bind – or their binding was significantly reduced – to an N-terminally truncated PrP variant (ΔF) with a deletion of amino acids 33–134, while it still bound to another variant (ΔE) with a shorter deletion of residues 33–121 (Supplementary Figure S4B). It remains unclear whether amino acids 121–134 are actively participating in the binding to POM4, 10, and 19, or if the deletion of this part induces a conformational change in the distant C-terminal part, which is certainly essential for the antibody-antigen interaction. Another possibility that we cannot formally exclude is that due to deletion of amino acids 33–134, ΔF is differentially glycosylated with a glycan chain that interferes with the epitope of these three POMs.

It is interesting that POM5, which was mapped to a loop region preceding the second α-helix of the globular domain of PrP^C^ (Figure 6), seems to immunoprecipitate PrP^Sc^ only after PK digestion (Figure 8A). This observation suggests that the POM5 loop epitope is potentially exposed on PrP^Sc^ only after truncation of the N-terminal part of the protein or, in other words, it is masked in the full-length PrP^Sc^ molecule.

Two of the N-terminal antibodies (POM2 and POM12) recognize four repetitive epitopes in the PrP sequence. Consequently, we could demonstrate that two antibody molecules can simultaneously bind one PrP molecule (Supplementary Figure S3B) [16]. Although we have not formally shown that the two binding sites of one IgG molecule of POM2 or POM12 attach concurrently to two epitope stretches within the octarepeat region of PrP, this represents the most plausible scenario. If this is indeed true, then the actual binding of the bivalent IgG POM2 and POM12 will be the result of this cooperative interaction. This property could well explain the fact that these two antibodies have consistently been among the most sensitive ones within our collection, despite the fact that the measured affinity of a single binding event of a monovalent POM2 (scFv) and the singular peptide epitope is only 20nM.

In conclusion, we present a new panel of high affinity monoclonal anti-PrP antibodies with specificities in diverse sites within the prion protein. With their comprehensive characterization in a variety of techniques, including western blotting, ELISA, flow cytometry, immunoprecipitation, immunohistochemistry and surface plasmon resonance, we think that the POMs are a very useful tool for prion research and diagnosis.

## Materials and Methods

### Antigen

Recombinant mouse PrP was produced in bacteria and purified as described in [24], [25]. Briefly, competent bacteria were transfected with a plasmid (pRSETA, Invitrogen) expressing N-terminally tagged (6 histidines) mouse PrP, either full length spanning amino acids 23–231 (rmPrP_23-231_) or N-terminally truncated, amino acids 121–231 (rmPrP_121-231_). PrP-expressing bacteria were grown until an OD of 1.0 was reached (6L of culture in LB) and protein expression was induced using isopropyl-beta-D-thiogalactopyranoside (IPTG). Bacteria were left to grow for another 5–7hrs and then harvested. Cells were resuspended in 6M Guanidium Hydrochloride, sonicated and centrifuged. The supernatant was run over a Ni-NTA column, which captures the 6his-tag attached to the N-terminus of PrP. A gradient buffer with a decreasing concentration of denaturing agent was passed over the immobilized PrP for refolding. Renatured PrP was then eluted using imidazole, which competes for the binding to Ni. The 6his-tag was removed by thrombin cleavage. Finally, the protein concentration was determined by its absorbance at 280nm and the purity after purification was checked by SDS-PAGE stained with Coomassie.

### Immunization

*Prnp^o/o^* mice were immunized with recombinant rmPrP_23-231_. For the initial injection, 10 µg of protein was emulsified in complete Freund's adjuvant (CFA). Boosting injections of 10–20 µg of protein subcutaneous (s.c.) or intravenous (i.v.) followed over a period of 65 days, according to the following schedule:

Day 1: s.c. injection of 10 µg rmPrP_23-231_+CFA, Day 22: s.c. injection of 10 µg rmPrP_23-231_, Day 43: s.c. injection of 20 µg rmPrP_23-231_, Day 64: i.v. injection of 10 µg rmPrP_23-231_, Day 65: i.v. injection of 10 µg rmPrP_23-231_, Day 66: i.v. injection of 10 µg rmPrP_23-231_, Day 66: Fusion.

### Fusion

After immunization, mice were sacrificed and spleen cells were isolated and fused with the immortalized myeloma cell line Sp2/0-Ag14 with polyethylene glycol (PEG) using a standard fusion protocol. The selection was made with HAT medium (Hypoxanthine, Aminopterin, Thymidine). Clones consisting of a small number of cells were transferred in fresh wells and cultured. Based on a first screen of 55 positive clones, 19 most interesting clones were selected for further characterization, hereafter termed POM1–19.

### Antibody purification

A standard antibody purification protocol was used (by GENOVAC GmbH, Germany). In brief, hybridoma cells were cultured to a log-phase state. During early scale up, standard FCS was step-wise exchanged with an ultra-low-IgG FCS while they were intensively checked for proliferation and viability. After scaling up, cells were transferred into a production flask in high density and then kept in static culture for a given period. Supernatant was harvested, centrifuged and filtered. Affinity purification was performed by binding to a Protein-G column followed by elution of the bound antibody with glycine buffer (pH = 3.0) and subsequent dialysis against PBS (pH7.2–7.4). Purity was checked by SDS-PAGE and protein concentration was determined by Lowry assay.

### Western Blotting

Equal amounts of recombinant prion proteins (100ng for Figure 5A and 10ng for Figure 5C) were mixed with loading dye and loaded on a 12% NuPAGE gel (Invitrogen, running with MES buffer). Mouse brain homogenates were prepared in PBS, 0.05% sodium deoxycholate, and 0.05% Nonidet P-40. For PK digestion 25 µg/ml of enzyme was used to digest PrP^C^. Samples corresponding to 40 µg of total proteins (with or without prior PK digestion) were mixed with loading dye and loaded on a 12% NuPAGE gel. Proteins were then transferred onto a nitrocellulose membrane incubated with hybridoma cell supernatant or purified monoclonal anti-PrP antibodies and horseradish peroxidase (HRP)-conjugated rabbit anti-mouse IgG (gamma) antibody (Zymed). For analysis of samples acquired from immunoprecipitation, blots were incubated with a biotinylated anti-PrP (POM1) antibody and HRP-labeled avidin (Pharmingen). Blots were finally incubated with HRP substrate (ECL, Pierce) and exposed on photosensitive film (Kodak) or with Versadoc 3000 imaging system (Bio-Rad).

### Anti-Native PrP^C^ Antibody Titer Determination

Splenocytes or blood cells derived from transgenic tg33 mice overexpressing PrP^C^ on T cells (Raeber et al., 1999b) were incubated with hybridoma cell supernatant of the novel monoclonal anti-PrP antibodies or with monoclonal anti-PrP antibody 6H4 (Prionics). Cells were washed and incubated with a FITC-labeled anti-mouse IgG secondary antibody or FITC-labeled anti-mouse IgM secondary antibody (Caltag Laboratories, Naenikon, Switzerland). Thereafter, cells were stained with phycoerythrin (PE)-labeled anti-Thy 1.2 for detecting T cells. All antibodies were obtained from Becton Dickinson unless otherwise indicated. Lysis of red blood cells was performed with FACS lysing solution (Becton Dickinson). Living cells were gated by using a combination of forward scatter and side scatter. Data were acquired on a FACS Calibur (Becton Dickinson) with CELLQUEST software (Becton Dickinson); post-acquisition analyses were performed with Windows Multiple Document Interface (WINMDI, version 2.8; http://facs.scripps.edu/).

### Peptides

Mouse PrP-peptide libraries were synthesized by Jerini, Germany. The first one consisted of 100 peptides, spanning the whole mature mouse PrP sequence. Each peptide was 12 amino acids long and their PrP-sequences were shifted by two amino acids each. The second one consisted of 25-mers, shifted by 5 amino acids each and spanned amino acids 111–225 of the mature mouse PrP sequence. As a control, a 25-mer containing a part of the octarepeat region – amino acids 65–89 – was used.

### ELISA

#### a. Direct ELISA against rmPrP for screening positive clones and for isotyping

Plates were coated at 4°C overnight with 5 µg/ml recombinant mouse PrP (either rmPrP_23-231_ or rmPrP_121-231_) diluted in PBS and were washed with PBS containing 0.1% (vol/vol) Tween-20 (PBST) They were blocked with 5% BSA and were then incubated for 2hrs at room temperature with 30 µl of 2-fold serially diluted hybridoma cell supernatant (1∶10 prediluted) in PBST containing 1–5% BSA. Plates were washed thoroughly with PBST and then probed with a horseradish peroxidase-conjugated anti-mouse antibody (total IgG, or isotype specific antibodies IgM, IgG_1_, IgG_2a_, IgG_2b_, IgG_3_ all from Zymed, used at 1∶1,000 dilution in 5% BSA, PBST). After washing, plates were developed with 2.2-azino-diethyl-benzothiazolinsulfonate, and optical density was measured at 405nm. Titer was defined as the highest dilution showing an OD more than two times the technical background, which was calculated as the average of uncoated wells and wells incubated without serum.

#### b. Competition ELISA for epitope mapping

384-well plates were coated with 300ng/ml rmPrP_23-231_ overnight at 4°C. Plates were washed with PBS containing 0.1% (vol/vol) Tween 20 (PBST), and blocked with 5% BSA for 2hrs at room temperature. After washing, plates were incubated with 30 µl of 2-fold serially diluted hybridoma cell supernatant (prediluted accordingly 1∶1000–1∶5000) in PBST containing 1% BSA with or without peptides in a final concentration of 0.8–8ng/ml. After 2hrs at room temperature, plates were washed 4 times and then probed with horseradish peroxidase conjugated rabbit anti-mouse IgG (1∶000 dilution, Zymed) for 1hr at room temperature. Plates were developed with 2.2-azino-diethyl-benzothiazolinsulfonate, and optical density was measured at 405nm. Titer was defined as the highest dilution showing an OD more than two times the technical background, which was calculated as the average of uncoated wells and wells incubated without serum.

#### c. Sandwich ELISA with biotinylated mAbs

96-well plates were coated with 400ng/ml of purified antibodies (designated POM1, 2, 3, 4 and 9) overnight at 4°C. Plates were washed with PBS containing 0.1% (vol/vol) Tween 20 (PBST), and blocked with 5% BSA for 2hrs at room temperature. After washing, plates were incubated with 50 µl of 2-fold serially diluted rmPrP_23-231_ in PBST containing 1% BSA, starting from a 100ng/ml dilution. After 1hr at room temperature, plates were washed extensively and then probed with biotinylated POMs at a concentration of 200ng/ml in PBST containing 1% BSA, for 1hr at room temperature. Subsequently, after washing, plates were incubated with horseradish peroxidase conjugated Avidin (1∶5000 dilution, Invitrogen) for 1hr at room temperature. Plates were developed with 2.2-azino-diethyl-benzothiazolinsulfonate (Boehringer), and optical density was measured at 405nm. Titer was defined as the highest dilution showing an OD more than two times the technical background, which was calculated as the average of uncoated wells and wells incubated without serum.

### SPR measurements

All experiments were performed with BIAcore3000 (Functional Genomics Center Zurich, FGCZ).

#### a. Pair wise mapping

Anti-PrP mAbs (POMs) or IgG_1_ for isotype control were immobilized on CM-5 chips (BIAcore) after 7 minute activation with NHS/EDC at a flow rate of 5 µl/min. 5 µl of a 50 µg/ml antibody solution in sodium acetate buffer pH = 4.5, were used to immobilize approximately 2500–5000 response units of covalently bound antibody. After inactivation of the surface with ethanolamine the surface was washed twice with SDS 0.5% (20 µl) to remove any non-covalently bound antibody. The system was primed and then a new sensogram was started with HBS-EP (BIAcore) as running buffer. 20 µl were used for all injections at a constant flow rate at 5 µl/min. Recombinant proteins or POM antibodies were injected on the chip (BSA or PrP) at a concentration of 50 µg/ml diluted in HBS-EP buffer. For regeneration of the chip several injections of SDS 0.5% (BIAcore) are needed until the baseline returns to 0. For control the injections were made to flow cells 1 and 2 or 3 and 4, where the first flow cell was coated with control IgG_1_ (Zymed) antibody and the second with each POM antibody.

#### b. Affinity measurements

BSA and rmPrP_121-231_ were covalently captured on flow cells 1 and 2 respectively of a CM5 chip (BIAcore) after activation with NHS/EDC at a flow rate of 5 µl/min. 5 µl of a 50 µg/ml antibody solution in sodium acetate buffer pH = 4.5, were used to immobilize approximately 1000 response units of covalently bound protein. After inactivation of the surface with ethanolamine, the system was primed and then a new sensogram was started with HBS-EP (BIAcore) as running buffer.

Antibodies were injected at a constant flow rate of 20 µl/min. All antibodies were diluted in HBS-EP buffer (BIAcore) in the following concentrations: 110nM, 54nM, 27nM, 13nM, 6.8nM, 3.4nM, 1.7nM, 850pM and 420pM. After the end of each injection, antibodies were allowed to dissociate for 20min (1200sec) and then the chip was regenerated with 100mM HCl (BIAcore). Sensograms in Supplementary Figure S7 represent changes in resonance units on flow cell immobilized with PrP, after subtraction of changes in resonance units on control flow cells (BSA).

For the equilibrium dissociation constant calculation with competition BIAcore, we first coupled a control (scrambled) or the POM2 epitope mimicking on flow cells 1 and 2 respectively of a CM5 chip (BIAcore) after activation with NHS/EDC as describe before. We then injected POM2 IgG (or scFv) diluted in HBS-EP buffer in the following concentrations: 12nM, 6nM, 4nM, 2nM, 1nm, 500pM, 250pM, 125pM and 0nM, with 100mM HCl after each concentration for regeneration. Each concentration was injected twice and the slope of the RU increase in the middle of each injection was used for creating the standard curve. We then prepared a 6nM POM2 scFv solution and mixed it with 13 different concentrations of the epitope-mimicking peptide, ranging from 100-0.01nM. The solutions were allowed to reach equilibrium at room temperature for 1hr and then concentration of free scFv POM2 in each tube was measured by BIAcore.

### Immunohistochemistry

Paraffin-fixed (1–2 µm) consecutive sections of mouse brains were deparaffinized by serial submersion in Xylene (3×5min), 100% ethanol (3×2min), 96% ethanol (3×3min) 70% ethanol (3min), distilled H_2_O and PBS. They were then submerged in citrate buffer (1.8mM Citric acid and 8.2mM Sodium citrate, pH = 10.6) and subjected to high pressure for 10min using a steam cooker. Samples were then allowed to cool down for 10min and then washed in PBS. They were then incubated for 10min in 3% H_2_O_2_ (in PBS) and washed in PBS. Slides were blocked for 1hr at room temperature (RT) with TNB buffer (0.10M Tris-HCl, 0.15M NaCl, pH = 7.5 with blocking reagent, Perkin Elmer) and then incubated at 4°C overnight with purified POM antibodies (2mg/ml, stock solutions) diluted 1∶500 on TNB. The next day, slides were washed with PBS and then incubated with an HRP-conjugated anti-mouse IgG antibody (Zymed) (1∶100 of 1mg/ml stock solution in TNB). After washing, slides were stained with a chromogenic HRP substrate AEC (DAKO) for approximately 20min at RT. Nuclear stain was performed with hematoxylin.

### Immunoprecipitation

Dynal tosylactivated M450 beads (Dynal No. 140.04) were covalently coupled with purified POM antibodies and M280 for plasminogen (Dynal No. 140.03). For the coupling reaction, 500 µl of beads were extensively washed in PBS and then resuspended in 1ml of coupling buffer (0.1M boric acid, pH = 9.5). 100 µg of purified Plasminogen or POM antibodies were added and the coupling reaction was performed with stark agitation at 37°C for 16hrs. After washing in PBS with 0.1% BSA, the reaction was stopped by incubating in 1ml of blocking buffer (0.2M Tris-HCl, pH = 8.5 with 0.1% w/v BSA) for 6hrs. 50 µg of brain proteins were diluted in 1ml of PAA buffer (PBS with 3%NP-40 and 3%Tween.20) with 20× protease inhibitors (Complete mini, Roche) and added on washed and magnetically collected beads without any liquid (corresponding to 10 µg of antibody coupled to 50 µl of beads). Beads were incubated with tilt rotation for 2hrs at RT and then washed extensively with PAA buffer. For the competition experiment, POM2, POM3 or POM6-coupled beads (corresponding to 5 µg of antibody coupled to 25 µl of beads) were preincubated for 1hr in RT with 1ml PAA with or without 40 µg/ml of peptides: QPHGGGW, for POM2 epitope or HNQWNK for POM3 epitope (negative control). Subsequently, 25 µg of brain proteins were added and incubated for 1hr at RT with rotation. Washed and magnetically collected beads were mixed with 20 µl of SDS-loading buffer. 10–30 µl of the protein solutions were used for western blot analysis.

### Construction, expression and purification of POM scFvs

#### a. Amplification and linking of VL and VH domains

RNA from each of the POM hybridomas (POM2, 11, 18, and 19) was diluted to 0.1 µg/µl and heated at 65°C for 10 minutes. After cooling the RNA samples on ice, 20 µL of each sample was used as a template for first strand complementary DNA (cDNA) synthesis using the First Strand cDNA synthesis kit (GE Healthcare Life Sciences, QC, Canada) according to the manufacturer's recommended procedure. The cDNA (5 µl) was used as a template for the polymerase chain reaction (PCR) amplification of the variable light (VL) and variable heavy (VH) antibody domains as described by [26] with modifications. The primers used to amplify the variable domains are listed below and were designed to allow for the incorporation of the scFv genes into the *SfiI* restriction enzyme sites of the pAK100 and pJB12 phage display vectors kindly supplied by Dr. Andreas Pluckthun, University of Zurich, Switzerland [27]. PCR reactions contained 150 µM dNTPs, 1.5mM Mg_2_^+^, Platinum Taq DNA polymerase (Invitrogen, ON, Canada) and reaction buffer used in the Expand High Fidelity PCR system (Roche Applied Science, QC, Canada). PCR products were analyzed by agarose gel electrophoresis and the amplified DNA was gel purified using the QIAEXII gel extraction kit (Qiagen, ON, Canada) according to the manufacturer's recommended procedure. A second round of PCR was performed in order to link the amplified VL and VH domains by splicing by overlap extension (SOE). This was accomplished by using 10ng of the VL and VH PCR products along with the ‘scforward’ and ‘scback’ primers (see below) in a PCR reaction using the same cycles and reaction conditions as before. The PCR products were analyzed by agarose gel electrophoresis and the scFv DNA (approximately 750 bp) was excised and gel purified as before.

#### b. Primers for PCR amplification of VL and VH domains

The listed sequences are written in the 5′ to 3′ direction and are provided using the IUPAC nomenclature of mixed bases (R = A or G, M = A or C, Y = C or T, K = G or T, S = C or G, W = A or T, H = A or C or T, B = C or G or T, V = A or C or G and D = A or G or T.)

Heavy chain forward primers:

HF1: GGAATTCGGCCCCCGAGGCCGAGGAAACGGTGACCGTGGT

HF2: GGAATTCGGCCCCCGAGGCCGAGGAGACTGTGAGAGTGGT

HF3: GGAATTCGGCCCCCGAGGCCGCAGAGACAGTGACCAGAGT

HF4: GGAATTCGGCCCCCGAGGCCGAGGAGACGGTGACTGAGGT

Heavy chain reverse primers:

HB1: GGCGGCGGCGGCTCCGGTGGTGGTGGATCCGAKGTRMAGCTTCAGGAGTC

HB2: GGCGGCGGCGGCTCCGGTGGTGGTGGATCCGAGGTBCAGCTGCAGCAGTC

HB3′: GGCGGCGGCGGCTCCGGTGGTGGTGGATCCCAGGTGCAGCTGAAGSARTC

HB4: GGCGGCGGCGGCTCCGGTGGTGGTGGATCCGAGGTCCARCTGCAACARTC

HB5: GGCGGCGGCGGCTCCGGTGGTGGTGGATCCCAGGTYCAGCTBCAGCARTC

HB6′: GGCGGCGGCGGCTCCGGTGGTGGTGGATCCCAGGTYCARCTGCAGCARTC

HB7′: GGCGGCGGCGGCTCCGGTGGTGGTGGATCCCAGGTCCACGTGAAGCARTC

HB8′: GGCGGCGGCGGCTCCGGTGGTGGTGGATCCGAGGTGAASSTGGTGGARTC

HB9′: GGCGGCGGCGGCTCCGGTGGTGGTGGATCCGAVGTGAWGSTGGTGGAGTC

HB10′: GGCGGCGGCGGCTCCGGTGGTGGTGGATCCGAGGTGCAGSTGGTGGARTC

HB11′: GGCGGCGGCGGCTCCGGTGGTGGTGGATCCGAKGTGCAMCTGGTGGARTC

HB12: GGCGGCGGCGGCTCCGGTGGTGGTGGATCCGAGGTGAAGCTGATGGARTC

HB13′: GGCGGCGGCGGCTCCGGTGGTGGTGGATCCGAGGTGCARCTTGTTGARTC

HB14′: GGCGGCGGCGGCTCCGGTGGTGGTGGATCCGARGTRAAGCTTCTCGARTC

HB15′: GGCGGCGGCGGCTCCGGTGGTGGTGGATCCGAAGTGAARSTTGAGGARTC

HB16′: GGCGGCGGCGGCTCCGGTGGTGGTGGATCCCAGGTTACTCTRAAASARTC

HB17: GGCGGCGGCGGCTCCGGTGGTGGTGGATCCCAGGTCCAACTVCAGCARCC

HB18′: GGCGGCGGCGGCTCCGGTGGTGGTGGATCCGATGTGAACTTGGAASARTC

HB19′: GGCGGCGGCGGCTCCGGTGGTGGTGGATCCGAGGTGAAGGTCATCGARTC

Light chain forward primers:

LF1′: GGAGCCGCCGCCGCC(AGAACCACCACCACC)2ACGTTTKATTTCCAGCTTGG

LF4: GGAGCCGCCGCCGCC(AGAACCACCACCACC)2ACGTTTTATTTCCAACTTTG

LF5: GGAGCCGCCGCCGCC(AGAACCACCACCACC)2ACGTTTCAGCTCCAGCTTGG

LFλ: GGAGCCGCCGCCGCC(AGAACCACCACCACC)2ACCTAGGACAGTCAGTTTGG

Light chain reverse primers:

LB1: GCCATGGCGGACTACAAAGAYATCCAGCTGACTCAGCC

LB2: GCCATGGCGGACTACAAAGAYATTGTTCTCWCCCAGTC

LB3: GCCATGGCGGACTACAAAGAYATTGTGMTMACTCAGTC

LB4: GCCATGGCGGACTACAAAGAYATTGTGYTRACACAGTC

LB5: GCCATGGCGGACTACAAAGAYATTGTRATGACMCAGTC

LB6: GCCATGGCGGACTACAAAGAYATTMAGATRAMCCAGTC

LB7: GCCATGGCGGACTACAAAGAYATTCAGATGAYDCAGTC

LB8: GCCATGGCGGACTACAAAGAYATYCAGATGACACAGAC

LB9: GCCATGGCGGACTACAAAGAYATTGTTCTCAWCCAGTC

LB10: GCCATGGCGGACTACAAAGAYATTGWGCTSACCCAATC

LB11: GCCATGGCGGACTACAAAGAYATTSTRATGACCCARTC

LB12: GCCATGGCGGACTACAAAGAYRTTKTGATGACCCARAC

LB13: GCCATGGCGGACTACAAAGAYATTGTGATGACBCAGKC

LB14: GCCATGGCGGACTACAAAGAYATTGTGATAACYCAGGA

LB15: GCCATGGCGGACTACAAAGAYATTGTGATGACCCAGWT

LB16: GCCATGGCGGACTACAAAGAYATTGTGATGACACAACC

LB17: GCCATGGCGGACTACAAAGAYATTTTGCTGACTCAGTC

LBλ: GCCATGGCGGACTACAAAGATGCTGTTGTGACTCAGGAATC

scforward primer:

GGAATTCGGCCCCCGAG

scback primer:

TTACTCGCGGCCCAGCCGGCCATGGCGGACTACAAAG

#### c. Cloning of scFv genes into phage display vectors

The PCR-amplified genes encoding scFvs for POM2, 11, 18, and 19 along with the pAK100 and pJB12 phage display vectors were digested with the *SfiI* restriction enzyme (New England Biolabs, ON, Canada) at 50°C overnight. The digested products were analyzed by agarose gel electrophoresis and gel purified as before. The gel purified digested scFv genes were ligated to the digested phage display vectors using the Rapid DNA ligation kit (Roche) according to the manufacturer's instructions. Competent TG1 *E. coli* cells (500 µl) were mixed with the ligation mixes (10–15 µl) and the solutions were incubated on ice for 45 minutes and then heat shocked in a 42°C waterbath for 2 minutes after which the samples were immediately placed on ice for 2 minutes. The cells were incubated in a 37°C shaker (250rpm) for 1 hour and then plated onto 2X YT agar plates supplemented with 1% (w/v) glucose and 30 µg/mL chloramphenicol. The plates were incubated at 37°C overnight to allow the growth of transformed cells.

#### d. Phage rescue and panning for isolation of antigen binders

The colonies (transformants) were resuspended in 5ml of 2X YT media supplemented with 1% glucose and 30 µg/mlchloramphenicol (2X YT-GC). Fresh 2X YT-GC media (5ml) was inoculated with 50 µl of resuspended colonies and the culture was incubated at 37°C at 250 rpm until the OD_600nm_ of the cultures was approximately 0.5. At this point, 5ml of fresh 2X YT-GC media containing 1mM IPTG and 1×10^9^ cfu VCSM13 helper phage (Stratagene, CA, USA) was added to the cultures, which were incubated overnight at 26°C with shaking (250rpm). The cultures were centrifuged (1000×g, 20 minutes, 22°C) and the supernatants containing the phage were collected. The supernatants containing the scFv-displaying phage were mixed with 2ml sterile PEG/NaCl solution (20% w/v PEG 8000 and 14.6% w/v NaCl), vortexed briefly and incubated on ice for 1 hour to precipitate them. The precipitated phage was collected by centrifugation (10,000×g, 20 minutes, 4°C) followed by the removal of the supernatant. The phage pellets were resuspended in 500 µl PBS containing BSA (2% w/v) and subsequently used to pan for the isolation of antigen binders as described below.

Wells of the ReactiBind Streptavidin-coated pre-blocked clear strip plates (Pierce, ON, Canada) were coated with 100 µl/well of 10 µg/ml biotinylated N-terminal peptide corresponding to residues 64–88 of the mouse PrP sequence (WGQPHGGSWGQPHGGSWGQPHGGGW) in PBS, pH = 7 for POM2, 11, and 18 and biotinylated C-terminal peptide corresponding to residues 202–226 (VKMMERVVEQMCVTQYQKESQAYYD) for POM19. PBS (100 µl/well) without the peptides was added to other wells to serve as uncoated controls. The plates were incubated at 22°C for 2 hours with agitation and then each well was washed 3 times with PBS. The phage suspension (100 µl/well) was added to the wells and the plates were incubated at 22°C for 2 hours with agitation. The plates were washed 20 times with sterile PBS containing 0.05% (v/v) Tween-20 followed by 20 additional washes with sterile PBS. The bound phage particles were eluted by the addition of sodium acetate buffer (100 µl; 0.1M acetic acid, 0.15M NaCl, pH = 2.8) and incubation for 8 minutes followed by neutralization with 12 µl 2M Tris buffer, pH = 9.5. The neutralized phage solutions were used immediately to infect 5ml log-phase TG1 *E. coli* cells (OD_600nm_ ∼0.5) for 30 minutes at 37°C followed by an additional 30 minutes at 22°C. The bacterial solutions were centrifuged (1000×g, 5 minutes, 4°C), 4ml of the supernatants were removed and the bacterial pellets were resuspended in the remaining liquid. The cells were spread evenly onto 2X YT-GC agar plates, which were incubated at 30°C overnight. The colonies were either used in a phage ELISA (see below) to identify antigen binders or they were pooled and used in another round of panning. 3–5 rounds of panning with decreasing concentrations of coating peptide in each round were usually required to isolate strong antigen binders.

#### e. Phage ELISA

After each round of panning, randomly selected colonies were used to inoculate separate 5ml of 2X YT-GC media. The cultures were incubated at 37°C, 250rpm until OD_600nm_ ∼0.5. Fresh 2X YT-GC media (5ml) supplemented with 1mM IPTG and 1×10^9^ cfu VCSM13 helper phage was added to each culture, which were then incubated overnight at 26–28°C, 250 rpm. The cultures were centrifuged (1000×g, 20min, 22°C) and the supernatants were added to separate 2ml of sterile PEG/NaCl to precipitate the phage. After vortexing, the samples were incubated on ice for 1 hour and then centrifuged (10,000×g, 20min, 4°C). The supernatants were discarded and the pellets were resuspended in 200 µl PBS containing BSA (2% w/v). ReactiBind Streptavidin-coated pre-blocked clear strip plates were coated with the appropriate peptide, incubated and washed with PBS as described above. Phage suspensions (100 µl/well) were added to the wells (coated and uncoated) and the plates were incubated at 22°C for 2 hours with agitation. The wells were washed 5 times with PBS containing 0.05% (v/v) Tween-20 followed by 6 times with PBS alone. 100 µl HRP/anti-M13 monoclonal conjugate diluted 1∶2500 (GE Healthcare Life Sciences) in PBS containing BSA (2% w/v) was added to each of the wells and the plates were incubated at 22°C for 1 hour with agitation. The wells were washed with PBS-Tween-20 and PBS alone as described above and then 100 µl of SureBlue Reserve TMB Microwell Peroxidase Substrate (KPL, MD, USA) was added to each well. The reaction was stopped by the addition of 1N HCl (100 µl/well) when satisfied with the color intensity and the absorbance was measured at 450nm. Clones were identified as strong antigen binders if the absorbance was at least ≥0.2 and at least ≥2 times the background. The DNA sequence for each of the strong antigen binders was determined and the corresponding amino acid sequences were aligned to identify differences in the sequences.

#### f. Expression and purification of POM2 scFv

The POM2 scFv gene for the strongest binder was subcloned into the bacterial expression vector pET30a (Novagen, WI, USA) at the *NdeI* and *XhoI* restriction enzyme sites of the multiple cloning site. Competent Rosetta (DE3) pLysS *E.coli* cells (Novagen) were transformed with the POM2 scFv-pET30a construct and used for bacterial overexpression for subsequent scFv purification. 150ml Luria-Bertani (LB) media containing 50 µg/ml kanamycin and 30 µg/ml chloramphenicol (LB-KC) was inoculated with a single colony of the transformed Rosetta (DE3) pLysS *E.coli*. The culture was incubated overnight at 37°C, 250rpm and 50ml of the overnight culture was used to inoculate 1L fresh LB-KC. The culture was incubated at 37°C, 250rpm until OD_600nm_ 0.4–0.6. IPTG (1mM final concentration) was added to the bacterial culture, which was then incubated overnight at 22°C, 250rpm or 3 hours at 37°C, 250rpm. The culture was centrifuged (15,000×g, 15min, 4°C) and the pellet resuspended in (30ml/L of culture) Buffer#1 (50mM NaH_2_PO_4_·H_2_O, 300mM NaCl, pH = 7.0). The sample was sonicated on ice to lyse cells and then centrifuged (15,000×g, 15 minutes, 4°C). Due to the expression of the POM2 scFv in the insoluble inclusion bodies, the supernatant was discarded and the pellet was resuspended in (30ml/L of culture) Buffer#2 (100mM NaH_2_PO_4_·H_2_O, 10mM Tris-HCl, 8M urea, 5mM imidazole, 5mM β-mercaptoethanol, pH = 8.0). The sample was stirred at 22°C for 1 hour and then centrifuged (10,000×g, 10min, 4°C). The supernatant was collected and added to (30ml/L culture) Ni-NTA agarose (Qiagen), which had been equilibrated with at least 10 column volumes of Buffer#2. After collecting the flow-through, the Ni-NTA agarose was washed first with approximately 5 column volumes of Wash Buffer#1 (100mM NaH_2_PO_4_·H_2_O, 10mM Tris-HCl, 8M urea, 10mM imidazole, pH = 8.0) and then with Wash Buffer#2 (100mM NaH_2_PO_4_·H_2_O, 10mM Tris-HCl, 8M urea, 250mM imidazole, pH = 8.0). The Wash Buffer#2 eluate containing POM2 scFv was collected in 40ml fractions and then analyzed by gel electrophoresis in a 13% SDS-PAGE gel stained with Coomassie Brilliant Blue R250 (Sigma-Aldrich, ON, Canada). To resolubilize the denatured purified POM2 scFv, the collected samples were dialyzed against 5mM Tris-HCl, pH = 7.5 at 4°C over 48 hours with multiple changes of dialysis buffer. The soluble POM2 scFv was lyophilized to concentrate and then resuspended in sterilized water to the desired protein concentration for subsequent experiments.

## Supporting Information

Figure S1 First screen and selection of best performing clones. Western Blot analysis of membrane strips containing PK-digested brain homogenate from terminally sick scrapie mice, using all 55 ELISA-positive clones. Blots were incubated with hybridoma cell supernatants and then with an HRP-labeled anti-mouse IgG secondary antibody. The red (POM) numbers indicate the 19 selected mAbs used for further characterization. Numbers on top indicate the individual clone numbers.
(9.96 MB TIF)

Figure S2 First screen and selection of best performing clones. Flow cytometric analysis of 55 clones using blood cells from mice overexpressing PrPC on T-cells. Blood cells were incubated with hybridoma cell supernatant or 6H4 and then incubated with a fluorescently labeled anti-mouse IgG antibody. Staining for a T-cell marker (CD3) allowed gating on T-cells and therefore analysis of PrP-specific binding. Red boxes indicate the 19 selected mAbs used in further characterization.
(7.56 MB TIF)

Figure S3 Sandwich ELISA with biotinylated POMs confirms SPR-results. (A)–(E) Each panel represents a 96-well plate incubated with one biotinylated POM as indicated. All plates were coated with unlabeled POM1, 2, 3, 4 and 9 as well as IgG1 for control. The results confirm that POM4 does not compete with POM1 or POM9 and that the N-terminal POM2 and POM3 do not compete with any of the C-terminal POM1 or POM9. At least two molecules of POM2 can bind simultaneously to one molecule of PrP.
(8.46 MB TIF)

Figure S4 Screening of POMs for binding to N-terminally truncated PrPs. (A) Equal amounts of brain proteins from wild type, ΔE-PrP (with a deletion of amino acids 33–121) with a wild type PrP allele, or two Prnpo/o mice were used on 12 replica blots, incubated with the indicated POMs. All N-terminal specific antibodies failed to recognize ΔE-PrP, confirming previous results. POM5 and POM7 showed a mono- and unglycosylated-specific binding pattern. (B) Replica blots with equal amounts of brain proteins from wild type, ΔE-PrP with a wild type PrP allele, ΔF-PrP (with a deletion of amino acids 33–134) or Prnpo/o mice incubated with the indicated POMs. POM4 and 10 (and POM19, not shown) did not recognize ΔF-PrP, suggesting that residues 121–134 are essential for the binding of these three antibodies. KO: Brain homogenate from Prnpo/o mice.
(8.16 MB TIF)

Figure S5 Binding of purified POM1–19 antibodies to cell-surface PrPC. Flow cytometric analysis comparing all POM antibodies to 6H4 for binding on PrPC-overexpressing T-cells. Interestingly, all N-terminal specific POMs show very strong binding to native PrPC as displayed on the surface of live cells.
(7.31 MB TIF)

Figure S6 Protein sequence alignment of scFvs from selected POMs and SDS-PAGE analysis of POM2 scFv purification. (A) The three CDRs of the VL and VH are highlighted in pink for CDR1, blue for CDR2 and green for CDR3. The amino acid linker between the VL and VH domains spans amino acids 108 to 128. The C-terminal Histidine tag was incorporated into each of the POM scFv sequences to enable purification by an affinity column. (B) Although there appears to be some POM2 scFv eluting from the column in the Flowthrough and the Wash fraction, pure POM2 scFv (∼27 kDa) is present primarily in the Elution fractions. The gel was stained with Coomassie Brilliant Blue R250.
(9.71 MB TIF)

Figure S7 Plasmon Resonance experiments and affinity determination of selected POMs. (A) SPR analysis of POM19 binding to immobilized rmPrP121 231. Purified POM19 was injected at different concentrations - ranging from 110nM to 420pM - and the binding constant was calculated with the BIAevaluation 3.1 software to be 870pM. (B) Affinity determination of scFv POM2 was done with competition SPR. First a standard curve of POM2 scFv with a panel of solutions at different antibody concentrations - ranging from 12nM–125pM - was created (left panel). Then, a series of pre-equilibrated solutions containing 6nM POM2 scFv and various concentrations of the epitope-mimicking peptide - 13 different concentrations, ranging from 100-0.01nM - were tested on the same chip in duplicates. We plotted the values of free antibody against those of the peptide competitor using the BIAevaluation software (right plot). The estimated equilibrium dissociation constant of the single binding event of scFv POM2 to the singular peptide epitope is 20nM.
(8.78 MB TIF)
