# Supplementary figures and images for: The POM Monoclonals: A Comprehensive Set of Antibodies to Non-Overlapping Prion Protein Epitopes
Source: PLoS One. 2008 Dec 8;3(12):e3872. doi: 10.1371/journal.pone.0003872 (PMC2592702; doi:10.1371/journal.pone.0003872)

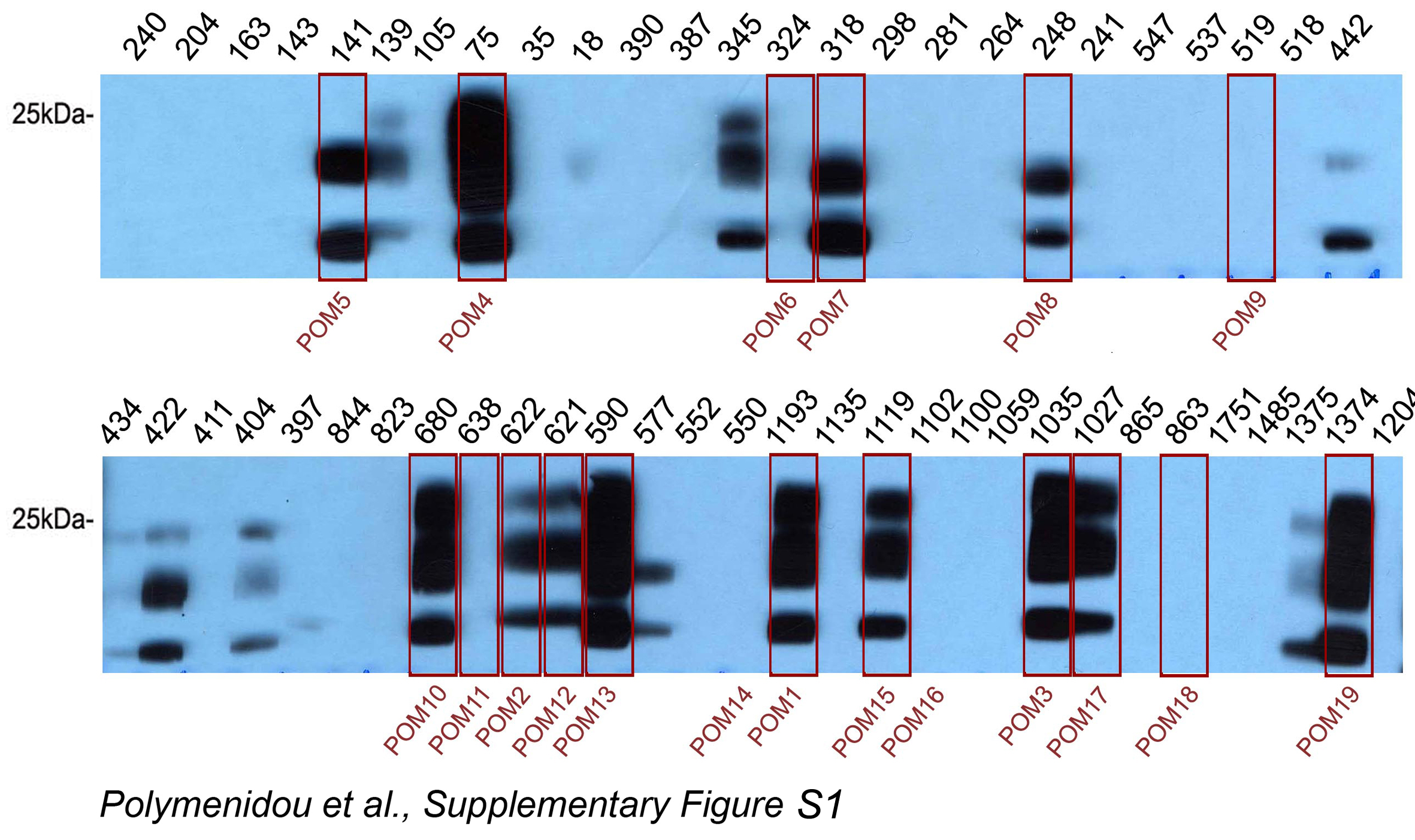

Supplement: Figure S1 — First screen and selection of best performing clones. Western Blot analysis of membrane strips containing PK-digested brain homogenate from terminally sick scrapie mice, using all 55 ELISA-positive clones. Blots were incubated with hybridoma cell supernatants and then with an HRP-labeled anti-mouse IgG secondary antibody. The red (POM) numbers indicate the 19 selected mAbs used for further characterization. Numbers on top indicate the individual clone numbers. (9.96 MB TIF) [file pone.0003872.s001.tif]

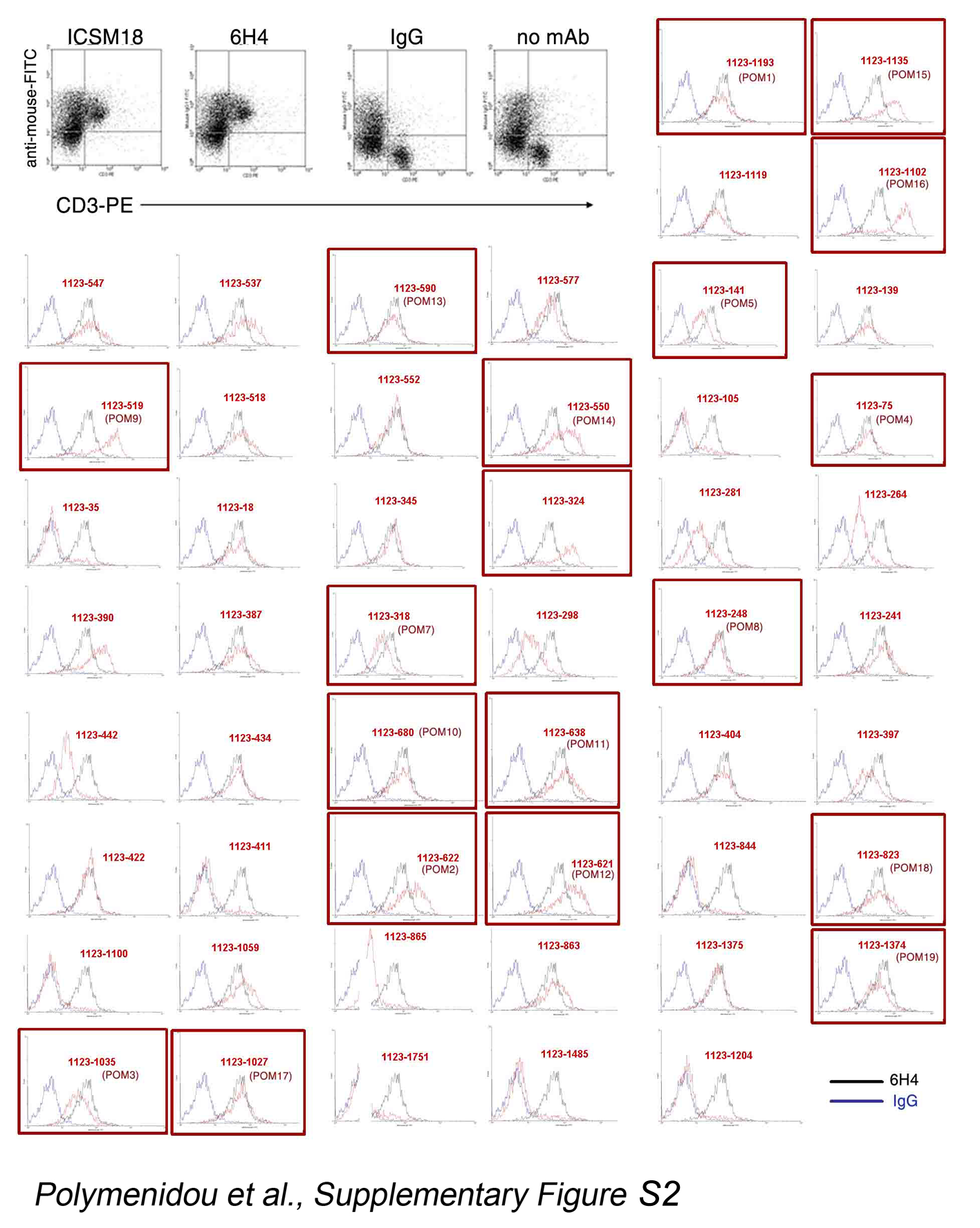

Supplement: Figure S2 — First screen and selection of best performing clones. Flow cytometric analysis of 55 clones using blood cells from mice overexpressing PrPC on T-cells. Blood cells were incubated with hybridoma cell supernatant or 6H4 and then incubated with a fluorescently labeled anti-mouse IgG antibody. Staining for a T-cell marker (CD3) allowed gating on T-cells and therefore analysis of PrP-specific binding. Red boxes indicate the 19 selected mAbs used in further characterization. (7.56 MB TIF) [file pone.0003872.s002.tif]

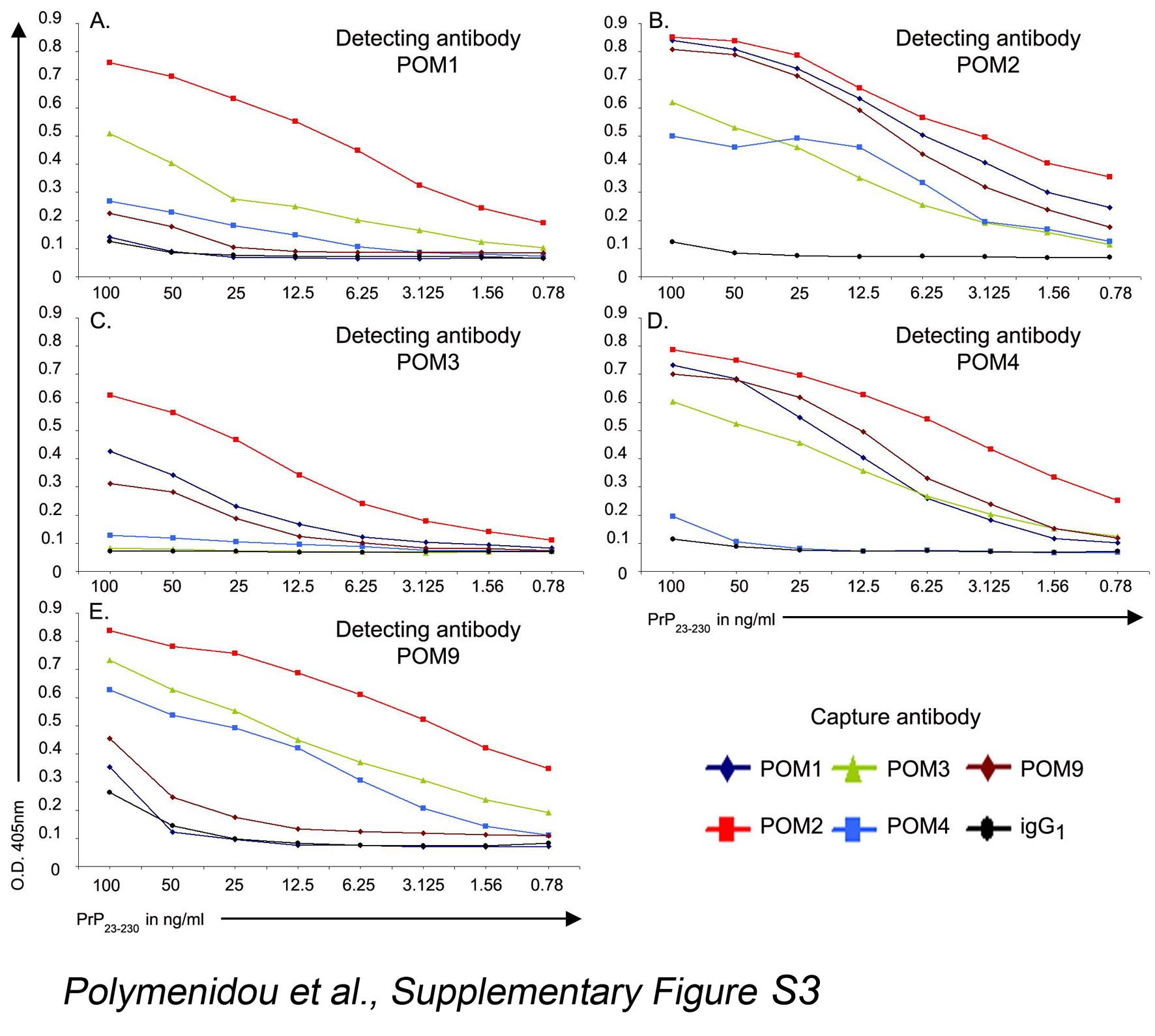

Supplement: Figure S3 — Sandwich ELISA with biotinylated POMs confirms SPR-results. (A)–(E) Each panel represents a 96-well plate incubated with one biotinylated POM as indicated. All plates were coated with unlabeled POM1, 2, 3, 4 and 9 as well as IgG1 for control. The results confirm that POM4 does not compete with POM1 or POM9 and that the N-terminal POM2 and POM3 do not compete with any of the C-terminal POM1 or POM9. At least two molecules of POM2 can bind simultaneously to one molecule of PrP. (8.46 MB TIF) [file pone.0003872.s003.tif]

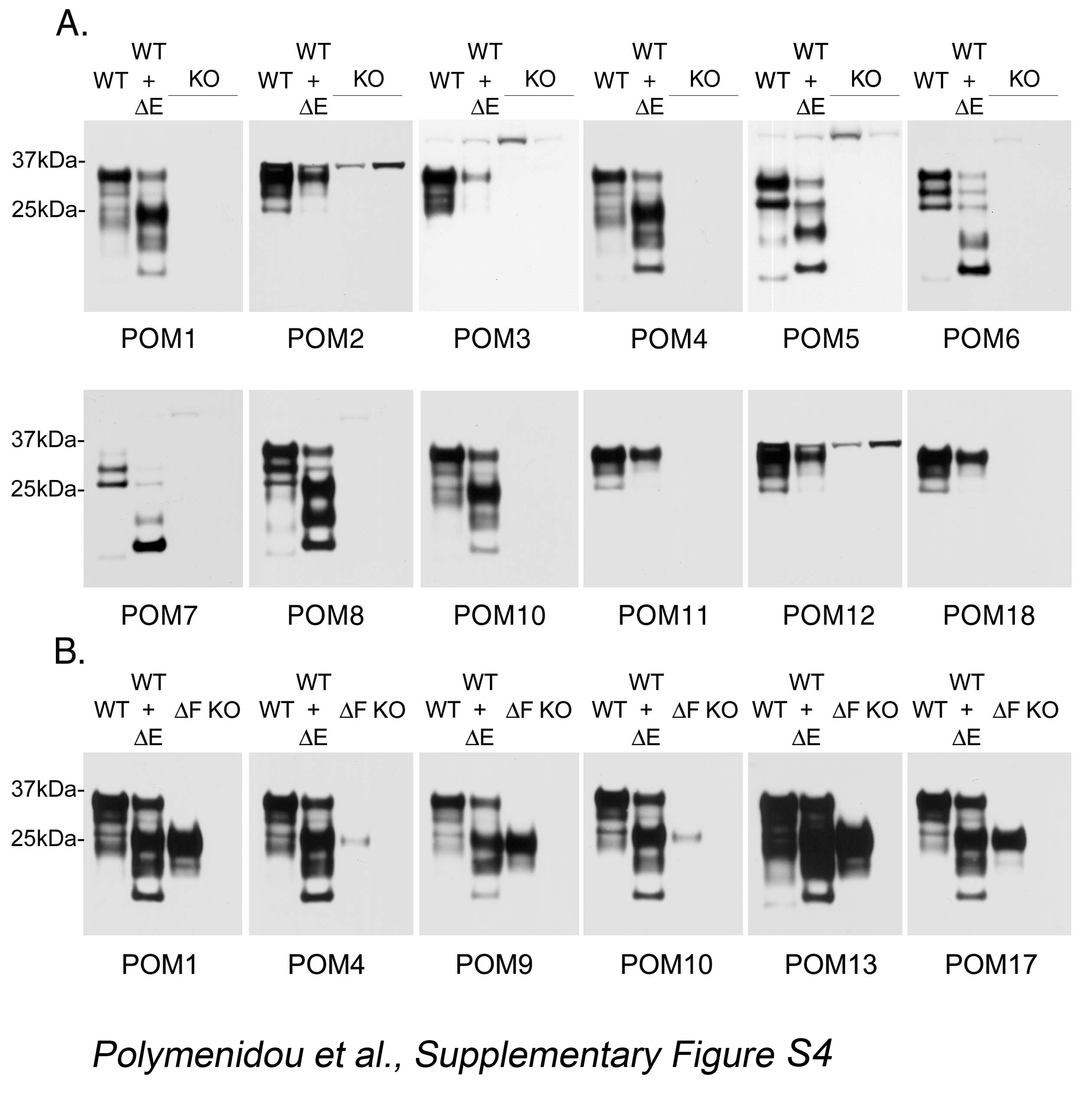

Supplement: Figure S4 — Screening of POMs for binding to N-terminally truncated PrPs. (A) Equal amounts of brain proteins from wild type, ΔE-PrP (with a deletion of amino acids 33–121) with a wild type PrP allele, or two Prnpo/o mice were used on 12 replica blots, incubated with the indicated POMs. All N-terminal specific antibodies failed to recognize ΔE-PrP, confirming previous results. POM5 and POM7 showed a mono- and unglycosylated-specific binding pattern. (B) Replica blots with equal amounts of brain proteins from wild type, ΔE-PrP with a wild type PrP allele, ΔF-PrP (with a deletion of amino acids 33–134) or Prnpo/o mice incubated with the indicated POMs. POM4 and 10 (and POM19, not shown) did not recognize ΔF-PrP, suggesting that residues 121–134 are essential for the binding of these three antibodies. KO: Brain homogenate from Prnpo/o mice. (8.16 MB TIF) [file pone.0003872.s004.tif]

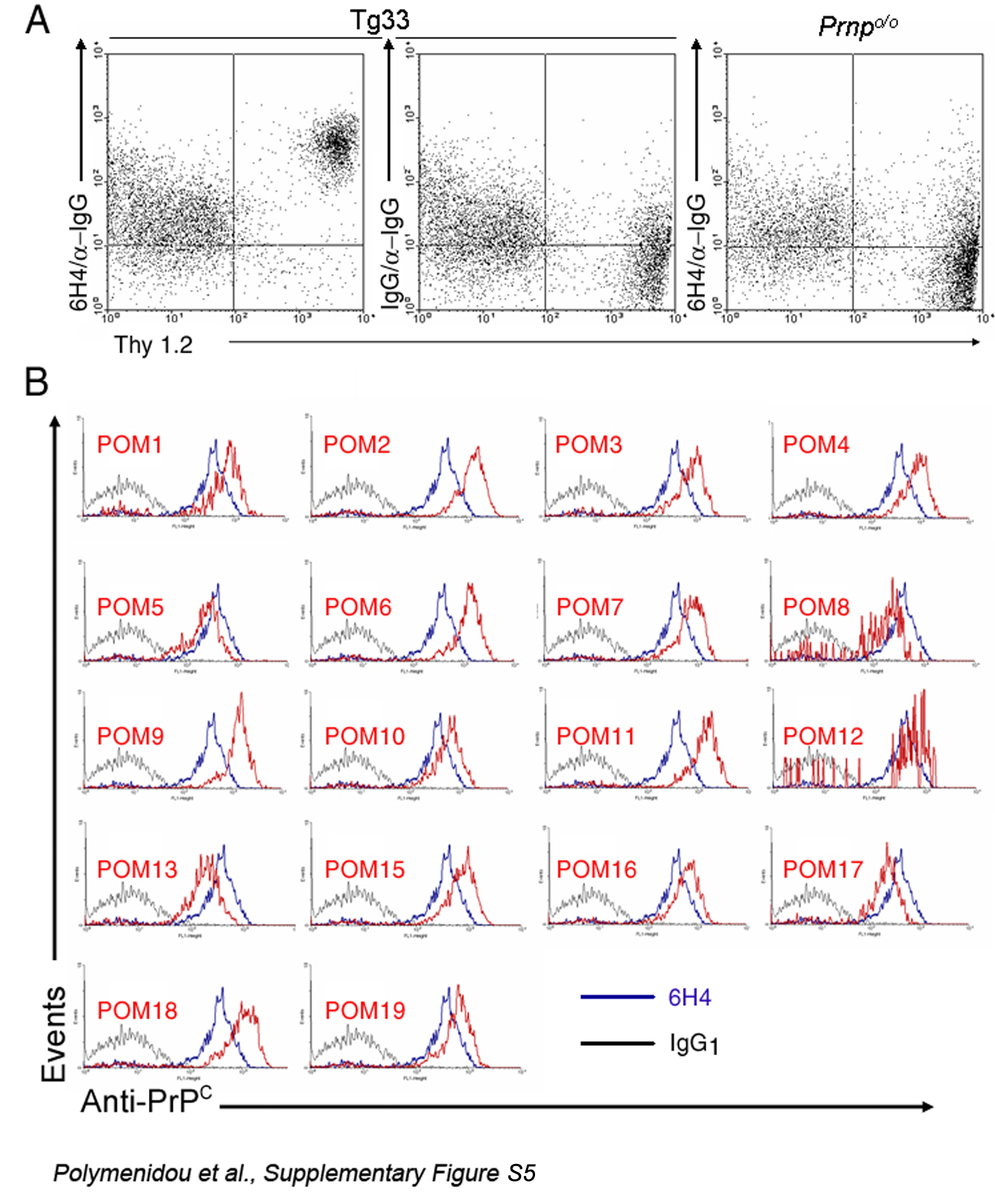

Supplement: Figure S5 — Binding of purified POM1–19 antibodies to cell-surface PrPC. Flow cytometric analysis comparing all POM antibodies to 6H4 for binding on PrPC-overexpressing T-cells. Interestingly, all N-terminal specific POMs show very strong binding to native PrPC as displayed on the surface of live cells. (7.31 MB TIF) [file pone.0003872.s005.tif]

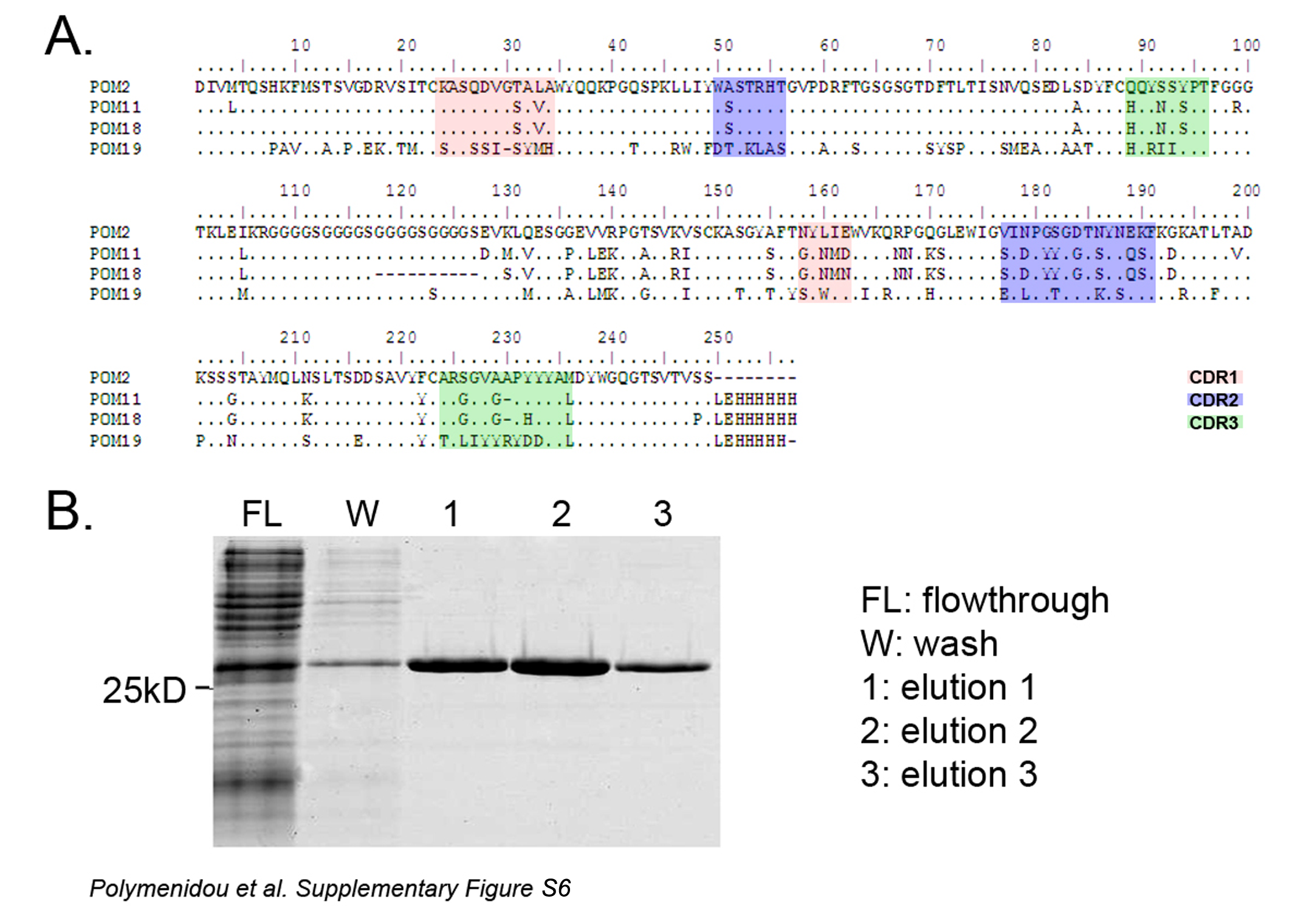

Supplement: Figure S6 — Protein sequence alignment of scFvs from selected POMs and SDS-PAGE analysis of POM2 scFv purification. (A) The three CDRs of the VL and VH are highlighted in pink for CDR1, blue for CDR2 and green for CDR3. The amino acid linker between the VL and VH domains spans amino acids 108 to 128. The C-terminal Histidine tag was incorporated into each of the POM scFv sequences to enable purification by an affinity column. (B) Although there appears to be some POM2 scFv eluting from the column in the Flowthrough and the Wash fraction, pure POM2 scFv (∼27 kDa) is present primarily in the Elution fractions. The gel was stained with Coomassie Brilliant Blue R250. (9.71 MB TIF) [file pone.0003872.s006.tif]

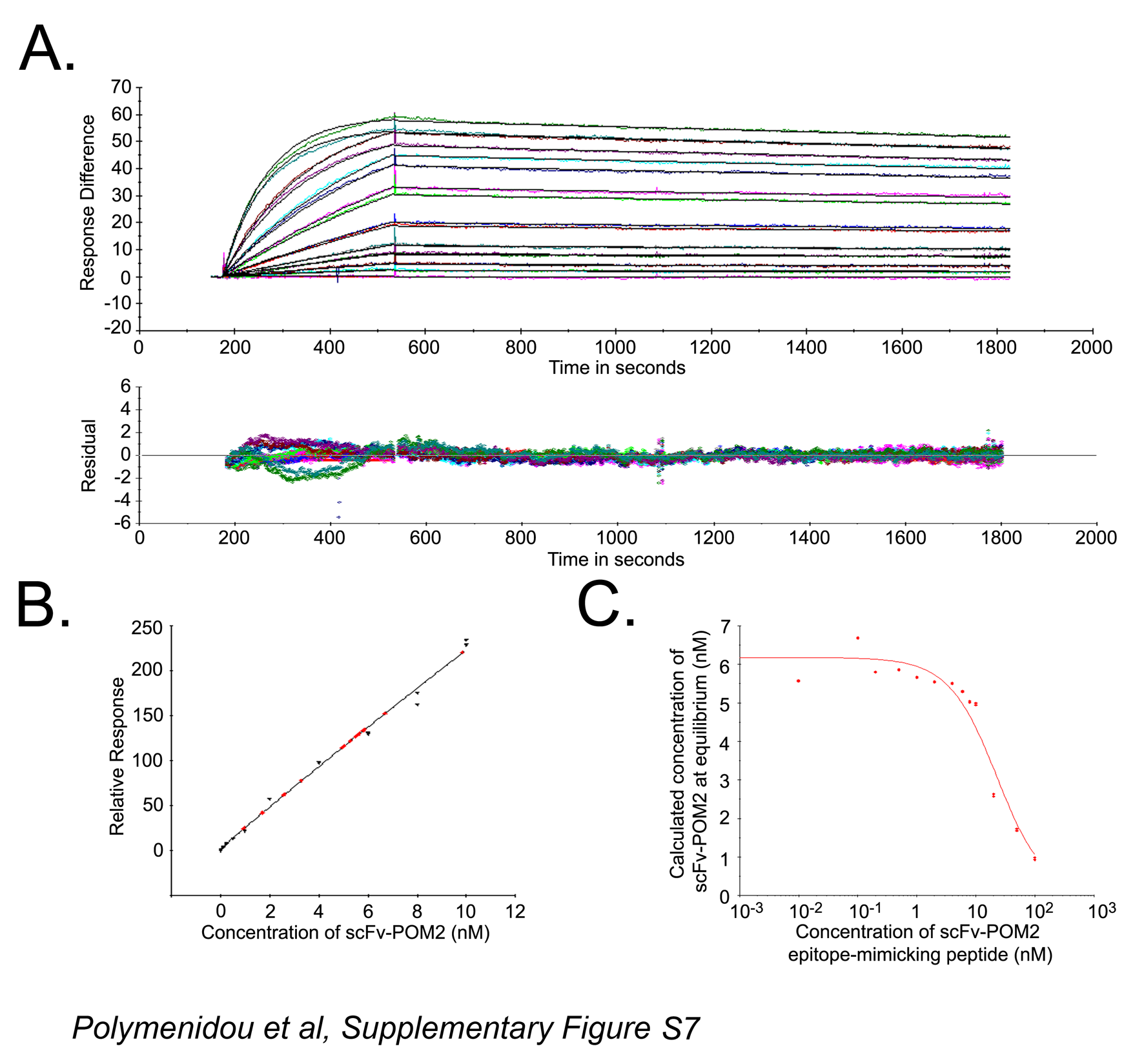

Supplement: Figure S7 — Plasmon Resonance experiments and affinity determination of selected POMs. (A) SPR analysis of POM19 binding to immobilized rmPrP121 231. Purified POM19 was injected at different concentrations - ranging from 110nM to 420pM - and the binding constant was calculated with the BIAevaluation 3.1 software to be 870pM. (B) Affinity determination of scFv POM2 was done with competition SPR. First a standard curve of POM2 scFv with a panel of solutions at different antibody concentrations - ranging from 12nM–125pM - was created (left panel). Then, a series of pre-equilibrated solutions containing 6nM POM2 scFv and various concentrations of the epitope-mimicking peptide - 13 different concentrations, ranging from 100-0.01nM - were tested on the same chip in duplicates. We plotted the values of free antibody against those of the peptide competitor using the BIAevaluation software (right plot). The estimated equilibrium dissociation constant of the single binding event of scFv POM2 to the singular peptide epitope is 20nM. (8.78 MB TIF) [file pone.0003872.s007.tif]
